# The emergence of new antidepressants for clinical use: Agomelatine paradox versus other novel agents

**DOI:** 10.1016/j.ibror.2019.01.001

**Published:** 2019-01-09

**Authors:** Olumuyiwa John Fasipe

**Affiliations:** Medical Lecturer and Senior Physician, Department of Clinical Pharmacology and Therapeutics, Faculty of Basic Clinical Sciences, University of Medical Sciences, Ondo City, Ondo State, Nigeria

**Keywords:** Emerging antidepressant agents, Depression disorders, Paradoxical agent

## Abstract

This study was designed with the rational aim of discussing the emerging antidepressant agents that are likely to bring positive landmark, tremendous improvement and significant impact to the management of patients with depression disorders. It also elaborates on the Agomelatine paradox vis-a-vis the other novel antidepressant agents. The emerging antidepressants are: selective monoamine oxidase inhibitors (MAOIs) such as bifemelane, pirlindole, toloxatone, selegiline, rasagiline and safinamide; serotonin-norepinephrine reuptake inhibitors (SNRIs) such as ansofaxine, nefopam and levomilnacipran; norepinephrine reuptake inhibitors (NRIs) such as Reboxetine, viloxazine, teniloxazine (also known as sulfoxazine or sufoxazine), and atomoxetine; Vilazodone (a serotonin 5-HT_1A_ autoreceptor partial agonist with serotonin reuptake inhibition [SPARI]); Vortioxetine (a serotonin receptors antagonist with serotonin reuptake inhibition [SARI]); atypical antipsychotics such as olanzapine, quetiapine, risperidone, lurasidone, aripiprazole and brexpiprazole; N-methyl-d-aspartate (NMDA)-glutamatergic neurotransmission system blockers such as ketamine, CP-101,606 (traxoprodil), GLYX-13 (rapastinel), NRX-1074 (Apimostinel) and Riluzole. While Agomelatine (a melatonergic MT**_1_** and MT**_2_** receptors agonist and a selective serotonergic 5-HT**_2B_** and 5-HT**_2C_** receptors antagonist [MASSA]) remains a paradoxical agent that doesn't fit into any of the currently available classes of antidepressant agents and its pharmacological properties also deemed it unfit and inappropriate to be classified into another separate novel class of antidepressants contrary to the reports published in previous reference literatures. Lastly, this review remarkably advocates for the incorporation of the atypical antipsychotics and NMDA-glutamatergic ionoceptor blockers as new member classes of the antidepressant agents because of their clinically significant roles in the management of depression disorders.

## 1 Introduction

The currently available antidepressants can be classified into thirteen different distinct classes based on their unique pharmacological mechanisms of action. As of this present moment, eleven (11) out of these thirteen (13) classes of antidepressants accomplish their pharmacological actions by blocking one or more of the reuptake transporter pumps and/or receptors for the three monoaminergic neurotransmitters, namely serotonin, norepinephrine and dopamine. The twelfth class inhibits the enzyme monoamine oxidase, while the thirteenth class works by blocking the NMDA-glutamatergic ionoceptor. This study was designed with the rational aim of discussing the emerging antidepressant agents that are likely to bring positive landmark, tremendous improvement and significant impact to the management of patients with depression disorders. It also elaborates on the Agomelatine paradox vis-a-vis the other novel antidepressant agents (Gelenberg et al., 2010; Mcintyre et al., 2017). The emerging antidepressants are: selective monoamine oxidase inhibitors (MAOIs) such as bifemelane, pirlindole, toloxatone, selegiline, rasagiline and safinamide; serotonin-norepinephrine reuptake inhibitors (SNRIs) such as ansofaxine, nefopam and levomilnacipran; norepinephrine reuptake inhibitors (NRIs) such as Reboxetine, viloxazine, teniloxazine (also known as sulfoxazine or sufoxazine), and atomoxetine; Vilazodone (a serotonin 5-HT_1A_ autoreceptor partial agonist with serotonin reuptake inhibition [SPARI]); Vortioxetine (a serotonin receptors antagonist with serotonin reuptake inhibition [SARI]); atypical antipsychotics such as olanzapine, quetiapine, risperidone, lurasidone, aripiprazole and brexpiprazole; N-methyl-d-aspartate (NMDA)-glutamatergic neurotransmission system blockers such as ketamine, CP-101,606 (traxoprodil), GLYX-13 (rapastinel), NRX-1074 (Apimostinel) and Riluzole (Gelenberg et al., 2010; Mcintyre et al., 2017; Gartlehner et al., 2016; Kasperet al., 2010;). While Agomelatine (a melatonergic MT**_1_** and MT**_2_** receptors agonist and a selective serotonergic 5-HT**_2B_** and 5-HT**_2C_** receptors antagonist [MASSA]) remains a paradoxical agent that doesn't fit into any of the currently available classes of antidepressant agents and its pharmacological properties also deemed it unfit and inappropriate to be classified into another separate novel class of antidepressants contrary to the reports published in previous reference literatures (Kasper et al., 2010; Heun et al., 2013; Stein et al., 2013; Koesters et al., 2013; Cipriani et al., 2018).

## 2 Classes of clinically available antidepressants

These different classes of clinically available antidepressants are: (Gelenberg et al., 2010; Mcintyre et al., 2017; Gartlehner et al., 2016)

1 Tricyclic antidepressants (TCAs) such as amitriptyline, imipramine, desipramine, nortriptyline, clomipramine, trimipramine, protriptyline and doxepin.
2 Monoamine oxidase inhibitors (MAOIs) such as phenelzine, nialamide, isocarboxazid, hydracarbazine, tranylcypromine, moclobemide, *bifemelane, *pirlindole, *toloxatone, *selegiline, *rasagiline and *safinamide.
3 Selective serotonin reuptake inhibitors (SSRIs) such as fluoxetine, sertraline, paroxetine, citalopram, escitalopram, and fluvoxamine.
4 Serotonin-norepinephrine reuptake inhibitors (SNRIs) such as venlafaxine, desvenlafaxine, duloxetine, *ansofaxine, *nefopam and *levomilnacipran.
5 Norepinephrine-dopamine reuptake inhibitor (NDRI) such as bupropion.
6 ^++^Selective norepinephrine reuptake inhibitors (NRIs) such as *Reboxetine, *viloxazine, *teniloxazine (also known as sulfoxazine or sufoxazine), and *atomoxetine.
7 Serotonin receptors antagonist with serotonin reuptake inhibition (SARI) such as trazodone, nefazodone, and *vortioxetine.
8 ^++^Serotonin 5-HT_1A_ autoreceptor partial agonist with serotonin reuptake inhibition (SPARI) such as *vilazodone
9 Noradrenergic α_2_ -receptor antagonist with specific serotonergic receptors-2 and -3 antagonism (NASSA) such as mirtazapine and ®mianserin.
10 ^++^Norepinephrine reuptake inhibitor with serotonin receptors antagonism (NRISA) such as maprotiline.
11 ^++^Serotonin-norepinephrine reuptake inhibitor and serotonin receptors antagonism antidepressant with potent antipsychotic D_2_ receptor blockade/antagonism (SNRISA with potent antipsychotic D_2_ receptor blockade/antagonism) such as amoxapine.
12 ^++^Atypical antipsychotics that exhibit weak D_2_ receptor antagonism with potently strong 5-HT_2A/2C_ receptor blockade such as *olanzapine, *quetiapine, *risperidone, *lurasidone, *aripiprazole and *brexpiprazole.
13 ^++^NMDA-glutamatergic ionoceptor blockers that exhibit a direct action on the excitatory glutamatergic neurotransmission system such as *ketamine, *CP-101,606 (traxoprodil), *GLYX-13 (rapastinel), *NRX-1074 (Apimostinel) and *Riluzole.

NOTE: ++Emerging antidepressant classes using mechanisms of action based classification; *Novel/emerging antidepressant drug(s) in a particular class; ®Drug approval was rejected/denied by the United States food drug administration (FDA) due to the submission of fraudulent data regarding its clinical trial by the investigators but had been approved for the treatment of depressive disorders long time ago in the European Union and other countries.

These emerging pharmacotherapeutic agents used for the treatment of depressive disorders are discussed below:

## 3 Agomelatine

### 3.1 Introduction of agomelatine as a paradoxical agent

Agomelatine belongs to the melatonergic MT**_1_** and MT**_2_** receptors agonist and selective serotonergic 5-HT**_2B_** and 5-HT**_2C_** receptors antagonism (MASSA) class. Concerning Agomelatine, as of this present moment and deeply analysing things from the psychopharmacological point of view; the utmost important question yet to be answered is "why should the drug -**Agomelatine** be regarded as an antidepressant agent when it did not actually possess the necessary pharmacoactivities and mechanism of actions that adequately qualified it to be classified under the family of antidepressants as done in previously published reference literatures?" (Kasper et al., 2010; Heun et al., 2013; Stein et al., 2013; Koesters et al., 2013; Cipriani et al., 2018) The mystery, approach and rationale behind this act of classification phenomenon were actually and inevitably putting a square peg inside a round hole; which is scientifically deemed unfit and inappropriate. This act of classification phenomenon makes Agomelatine to be referred to as a paradoxical agent that contradicts itself. Furthermore, drug like Cyproheptadine is potently a strong antagonist at the serotonergic 5-HT**_2A_**, 5-HT**_2B_** and 5-HT**_2C_** receptors, a strong antagonist/inverse agonist at the histaminergic H**_1_** receptor and also exhibits a moderate unselective blockade/antagonism at the muscarinic acetylcholine [M] receptors. Nevertheless, Cyproheptadine is acceptable as an anxiolytic-sedative agent but is not worthy to be regarded and classified as an antidepressant agent based on these pharmacological properties. In addition, drug like Ramelteon or Tasimelteon is a melatonergic MT**_1_** and MT**_2_** receptors agonist used for the treatment of non-24-hour sleep–wake rhythm disorder (also called Non-24, N24 and N24HSWD). Yet, Ramelteon or Tasimelteon is acceptable as a sedative agent but is not worthy to be regarded and classified as an antidepressant agent based on these pharmacological properties. Hence, in a nutshell, why should Agomelatine (a melatonergic MT**_1_** and MT**_2_** receptors agonist and a selective serotonergic 5-HT**_2B_** and 5-HT**_2C_** receptors antagonist [MASSA]) be given a separate and different preferential treatment from Cyproheptadine, Ramelteon or Tasimelteon in the actual medical context? This implies that what is sauce for the goose is also sauce for the gander in the real sense! Furthermore, to buttress this point of view, the NMDA-glutamatergic ionoceptor blockers such as rapastinel, apimostinel and ketamine produce their rapid and sustain antidepressant activity through a novel mechanism of action that involve the inhibition/blockade of the NMDA-glutamatergic ionoceptor which are unchallengeable and clinical obvious, in contrast to Agomelatine (MASSA) whose claimed antidepressant activity is quite ambiguous and highly questionable when used solely as a monotherapy for the treatment of depression disorders because of inadequate clinical efficacy and response in particular ! (Stein et al., 2013; Koesters et al., 2013; Cipriani et al., 2018)

Agomelatine was discovered and developed by the European pharmaceutical company Servier Laboratories Limited. Servier developed the drug and conducted its phase III trials in the European Union. In March 2005, Servier submitted agomelatine to the European Medicines Agency (EMA) for licencing and marketing approval. On 27th July 2006, the Committee for Medical Products for Human Use (CHMP) of the EMA recommended a refusal of the marketing authorisation. The major concern was that efficacy had not been sufficiently shown, while there were no special concerns about side effects. Again, in September 2007, Servier submitted a new marketing application to the EMA. In March 2006, Servier announced it had sold the rights to market Agomelatine in the United States (US) to Novartis. It was undergoing several phase III clinical trials in the US, and until October 2011 Novartis listed the drug as scheduled for submission to the Food Drug Administration (FDA) no earlier than 2012. However, the development for the US market was discontinued and withdrawn in October 2011, when the results from the last of those trials became available. It received EMA approval for marketing in the European Union in February 2009 and Therapeutic Goods Administration (TGA) approval for marketing in Australia in August 2010 (Heun et al., 2013; Stein et al., 2013; Koesters et al., 2013; Cipriani et al., 2018).

### 3.2 Pharmacological properties of agomelatine

Agomelatine is a melatonergic MT**_1_** and MT**_2_** receptors agonist and a selective serotonergic 5-HT**_2B_** and 5-HT**_2C_** receptors antagonist (MASSA). The 5-HT**_2B_** receptors are poorly represented in the central nervous system (CNS) in contrast to the 5-HT**_2C_** receptors. These 5-HT**_2B_** receptors are found predominantly in the periphery on platelets, and endothelial lining of the heart valves and blood vessels in the cardiovascular system. Binding studies indicate that it has no effect on monoaminergic reuptake transporter pumps and no affinity for noradrenergic, histaminergic, cholinergic, dopaminergic, glutamatergic, benzodiazepine receptors nor other serotonergic receptor subtypes (Kasper et al., 2010). Agomelatine prochronobiological activity resynchronises and entrains circadian rhythm activity in experimental animal models of delayed sleep phase syndrome via its melatonergic MT**_1_** and MT**_2_** receptors agonistic effect, via inducing a phase advance of sleep by reducing the duration of sleep latency period. In humans, the MT**_1_** receptors are expressed in the pars tuberalis and pars distalis of the anterior pituitary gland and suprachiasmatic nuclei of the hypothalamus where they mediate and control reproductive physiological function and melatonin’s biological circadian rhythm activity, respectively. While the MT**_2_** receptors are expressed in the retina and osteoblasts. These MT**_2_** receptors' expression in the retina is indicative of melatonin's effect on the mammalian retina occurring through this receptor. Activation of melatonin MT**_2_** receptors in the retina has been found to affect and delay several light-dependent functions, including phagocytosis and photopigment disc shedding. Also MT**_2_** receptor regulates proliferation and differentiation of osteoblasts and further enhances their osteogenic function in depositing new bone matrices (Heun et al., 2013). Agonist activation of the pertussis toxin‐sensitive inhibitory G‐protein coupled (G**_i/o_**) melatonergic MT**_1_** and MT**_2_** receptors leads to the inhibition of adenylyl cyclase and guanylyl cyclase activities respectively, with subsequent reduction of intracellular cAMP and cGMP second messengers, respectively (Stein et al., 2013). By antagonizing the neocortical postsynaptic serotonergic 5-HT**_2C_** receptors, Agomelatine disinhibits/increases norepinephrine and dopamine release specifically in the neocortical areas such as the prefrontal cortex but neither in the subcortical areas such as the striatum nor nucleus accumbens. Therefore, it is sometimes referred to as a **norepinephrine–dopamine disinhibitor (NDD)** (Heun et al., 2013; Koesters et al., 2013; Cipriani et al., 2018). It also worth mentioning here that dopaminergic and adrenergic neurotransmission pathways in neocortical areas such as the prefrontal cortex, entorrhinal cortex, cingulate cortex, superior temporal cortex and orbital cortex are hypofuctionally impaired in depression disorders. Agomelatine has no influence on the extracellular levels of serotonin. It has been postulated to exhibit an antidepressant-like effect in experimental animal models of depression (learned helplessness test, despair test, chronic mild stress) as well as in models with circadian rhythm desynchronisation disorder type 1 (CRDD-1) and in models related to stress, insomnia and anxiety (Heun et al., 2013; Koesters et al., 2013; Cipriani et al., 2018). Infact, it has been demonstrated that genetically modified knock-out experimental model mice lacking 5-HT**_2C_** receptors significantly exhibit/manifest reduced and limited anxiety symptoms. Hence, by antagonizing the postsynaptic serotonergic 5-HT**_2C_** receptor in the subcortical areas such as basal ganglia, mesolimbic cortex and hippocampus; Agomelatine produces anxiolytic effect clinically (Kasper et al., 2010; Heun et al., 2013; Koesters et al., 2013). In humans, agomelatine has positive phase shifting properties; it induces a phase advance of sleep by reducing the duration of sleep latency period, body temperature decline and melatonin-like onset of action/activity. From the psychopharmacological point of view, agomelatine will be efficacious as an adjunct-augmenting pharmacotherapeutic agent for the treatment of patients having anxious depression disorders (that is, either major depression disorder [MDD] or bipolar depression or schizoaffective depression with anxiety disorder component). It will also be efficacious as a sole or combine pharmacotherapeutic agent for the treatment of patients having delayed sleep phase syndrome due to circadian rhythm desynchronisation disorder type 1 (CRDD-1) or Jetlag dysrhythmia, insomnia, anxiety disorders, selective serotonin reuptake inhibitor (SSRI)-induced sexual dysfunction and/or SSRI-induced nocturnal myclonus/akathisia (Kasper et al., 2010; Heun et al., 2013; Stein et al., 2013; Koesters et al., 2013; Cipriani et al., 2018). In circadian rhythm desynchronisation disorder type 1 (CRDD-1), there is deficiency of melatonin production as a result of lesional destruction of the pinealocytic neurons in the pineal gland. While circadian rhythm desynchronisation disorder type 2 (CRDD-2) occurs as a result of lesional destruction of the suprachiasmatic nuclei or loss of function mutation affecting the melatonergic MT**_1_** receptors on the suprachiasmatic nuclei in the hypothalamus. It also worthy of note that the suprachiasmatic nucleus function as the chronobiological clock of the human body and any disruption in its functional activity will inevitably affect the circadian (sleep-wake) rhythm cycle (Kasper et al., 2010; Stein et al., 2013; Koesters et al., 2013). Agomelatine alone may not be effective as a monotherapy for the treatment of unipolar depression or bipolar depression or schizoaffective depression because of its unique mechanism of action as a melatonergic MT**_1_** and MT**_2_** receptors agonist and a selective serotonergic 5-HT**_2C_** receptor antagonist (MASSA) (Heun et al., 2013; Stein et al., 2013; Koesters et al., 2013; Cipriani et al., 2018). Because Agomelatine lacks inhibitory pharmacoactivity at the monoaminergic reuptake transporter pumps (SERT, NET and DAT), does not inhibit the enzyme monoamine oxidase, has neither weak antagonist nor partial agonist activity at the dopaminergic D**_2_** receptor, and also lacks antagonistic activity at both the noradrenergic α_2_ -receptor and NMDA-glutamatergic ionoceptor; the author of this review propose and recommend that Agomelatine should not be regarded and accepted as an antidepressant, but rather, it should be classified as an anxiolytic-sedative agent on account of its melatonergic MT**_1_** and MT**_2_** receptors agonist and selective serotonergic 5-HT**_2C_** receptor antagonistic (MASSA) properties. Moreover, Agomelatine remains a paradoxical agent that doesn't fit into any of the currently available classes of antidepressant agents and its pharmacological properties also deemed it unfit and inappropriate to be classified into another separate novel class of antidepressants contrary to the reports published in previous reference literatures (Kasper et al., 2010; Heun et al., 2013; Stein et al., 2013; Koesters et al., 2013; Cipriani et al., 2018). The claimed but ambiguous and questionable antidepressant activity of Agomelatine in some documented previous reference literatures could be as a result of its sedative action via the melatonergic MT**_1_** and MT**_2_** receptors agonism with its anxiolytic action via the 5-HT**_2C_** receptor antagonism. This could easily be misinterpreted through a bias cloudy observation as a mild antidepressant effect clinically because some patients with depression disorders tend to present with complains/features of insomnia and anxiety which Agomelatine can help alleviate to some extent and they will feel better with these improvements (Kasper et al., 2010; Heun et al., 2013; Stein et al., 2013; Koesters et al., 2013; Cipriani et al., 2018). Furthermore, according to the results obtained from the systematic review and meta-analysis study conducted by Cipriani et al., (2018), the questionable antidepressant activity and effect claimed to be exhibited by Agomelatine among a variety of antidepressants in the acute treatment of adults with unipolar major depressive disorder in their assessment of previously published and unpublished studies (Kasper et al., 2010; Heun et al., 2013; Stein et al., 2013; Koesters et al., 2013; Cipriani et al., 2018) could also be attributed to the investigators bias as the pharmaceutical company that produces Agomelatine is quite desperate to market the drug as an antidepressant agent rather than marketing it as an anxiolytic-sedative agent. Also the study by Cipriani et al., (2018) was funded by the National Institute for Health Research Oxford Health Biomedical Research Centre, and in conjunction with the Japan Society for the Promotion of Science. But this particular study do not receive funding from anyone and is highly predisposed to say the truth even if there is going to be vivid criticisms, rejections and disputations. In addition, the Cipriani et al., (2018) study completely excluded the participants with bipolar depression, psychotic depression, or treatment-resistant depression; once again, if these researchers were quite very sure of the antidepressant activity of Agomelatine for these excluded disease conditions, these excluded patients with bipolar depression, psychotic depression, or treatment-resistant depression should have been included and considered since depression disorder was a significant component of their pathological conditions, as this deliberate action also justified their uncertainty concerning the efficacy of Agomelatine alone monotherapy for these depression disorders. One truthful fact the pharmaceutical company producing Agomelatine is ignorance/unaware of today is that, if this drug is marketed primarily as an anxiolytic-sedative agent, this does not stop clinicians from using it as an adjunct-augmenting pharmacotherapeutic agent for the treatment of depression disorders. From clinical evidences and based on psychopharmacological stand point of view, it will be highly advisable for the pharmaceutical company producing Agomelatine to market it primarily as an anxiolytic-sedative agent; so that the drug can quickly be approved by the FDA, to be widely accepted by the clinicians, and at the same time still being used secondarily (or off-label) as an adjunct-augmenting pharmacotherapeutic agent for the treatment of anxious depression disorders (Fasipe et al., 2018).

In addition, agomelatine use was not associated with discontinuation or withdrawal symptoms after an abrupt/sudden cessation of treatment after 12 weeks duration of pharmacotherapy. It has a mean terminal half-life of about 2 h 20 min (140 min). After oral administration, agomelatine is rapidly (0.5–4 h) and well absorbed (80%) and the time at which maximum blood concentration was achieved was between 45 min and 90 min after a single oral dose of 25–50 mg. However, its bioavailability is low at the therapeutic oral dose due to the high first-pass metabolism, which may be of concern especially in elderly patients over 75 years or in subjects with hepatic compromise or renal impairment. It has a moderate volume of distribution of approximately 35 L, a plasma protein binding of 95%, and the peak plasma concentration is achieved within 1–2 h after of oral administration. At the therapeutic levels, agomelatine blood concentration increases proportionally with dose; at higher doses, a saturation of the first-pass effect may occur. About 80% of the drug is eliminated through urinary excretion of the metabolites, whereas a small amount of the metabolites undergoes fecal excretion. The major enzymes involved in the biotransformation of agomelatine are CYP1 A2 (90%), and to a lesser extent, CYP2C9/CYP2C19 (Stein et al., 2013; Koesters et al., 2013).

## 4 The emerging glutamatergic hypothesis of depression

**Fig. 1:**
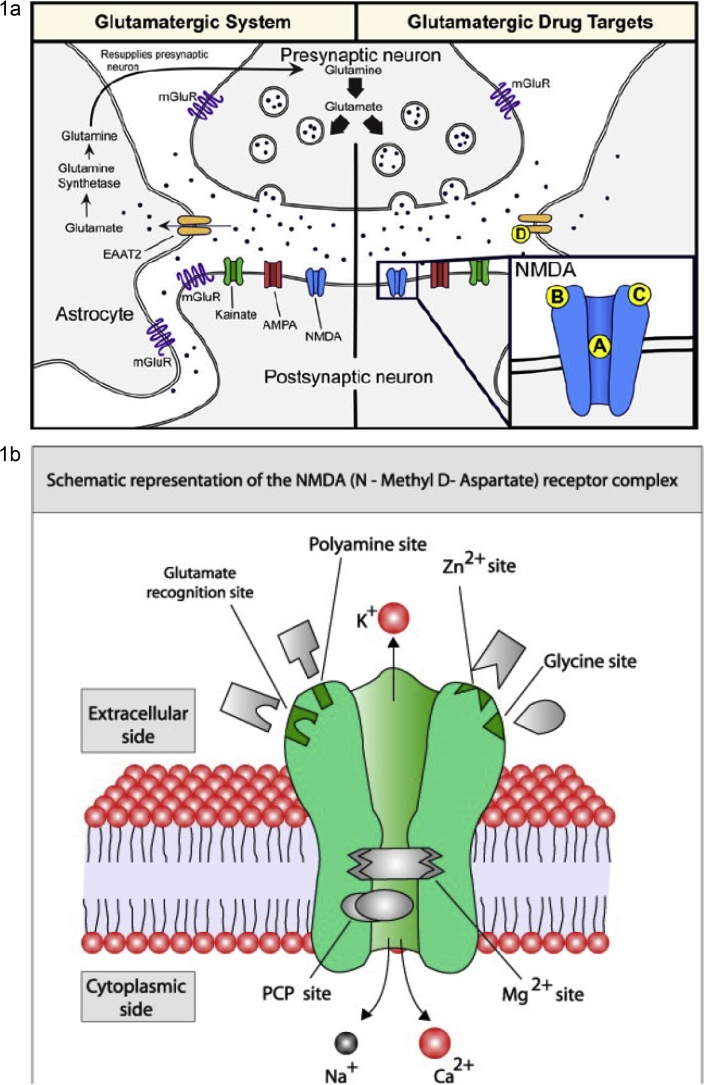
a showed the tripartite glutamatergic synapse and potential drug targets. *Left panel*: The presynaptic neuron releases glutamate neurotransmitter in response to action potentials. The glutamate neurotransmitter can bind to ionotropic (i.e. NMDA, AMPA, kainate) and metabotropic (i.e mGluR) receptors located on the presynaptic and postsynaptic neuron as well as on astrocytes. Synaptic glutamate reuptake is performed primarily by the EAAT-2 located on astrocytes. Within the astrocyte, glutamate is converted to glutamine (glutamate/glutamine cycle) via glutamine transaminase (synthetase) and then resupplied to the presynaptic neuron where it is used for the biosynthesis of glutamate neurotransmitter. *Right panel*: Potential NMDA and EAAT-2 drug targets: (A) Noncompetitive GluN2 subunits-unselective NMDA receptor antagonists (e.g. ketamine and memantine) and low-trapping NMDA receptor channel blockers (lanicemine [AZD6765]); (B) GluN2B subunit-selective NMDA receptor antagonists (e.g. traxoprodil [CP-101,606] and MK-0657); (C) GluN1 subunit-selective NMDA receptor partial agonists (e.g. GLYX-13 [Rapastinel], NRX-1074 [Apimostinel], and D-cycloserine); (D) EAAT-2 reuptake enhancer (e.g. Riluzole). Abbreviations: NMDA, N-methyl-D-aspartate; AMPA, α-amino-3-hydroxy-5-methyl-4-isoxazolepropionic acid; mGluR, metabotropic glutamate receptors; EAAT-2, excitatory amino acid transporter-2. b showed a schematic representation of the NMDA-glutamatergic receptor (NMDAR) heteromeric complex.

Before thoroughly discussing the NMDA-glutamatergic ionoceptor blockers (antagonist/inverse agonist/partial agonist) as a separate novel class of the antidepressant agents, let concisely look at the emerging glutamatergic hypothesis of depression disorders in full details. In the central nervous system, glutamate is the major excitatory neurotransmitter and makes functional contributions to more than half of all the synapses in the brain. The glutamate system has an integrated tripartite synapse that consists of: (1) a presynaptic neuron, (2) a postsynaptic neuron, and (3) an astrocyte. Fig. 1a showed the tripartite glutamatergic synapse and potential drug targets. The presynaptic neuron releases glutamate in response to action potentials. The released glutamate then binds to various pre- and postsynaptic receptors, as well as to receptors on the surrounding astrocytes. Synaptic glutamate reuptake is performed primarily by astrocytes, specifically, the excitatory amino acid transporter-2 (EAAT-2). Within the astrocyte, glutamate is converted to glutamine (glutamate/glutamine cycle) by glutamine synthetase and then resupplied to the presynaptic neuron where it is used for synthesis of glutamate. The glutamatergic system consists of two receptor types namely, ionotropic and metabotropic receptors. The Ionotropic glutamatergic receptors include N-methyl-d-aspartate (NMDA) receptors, α-amino-3-hydroxy-5-methyl-4-isoxazole propionic acid (AMPA), and kainate receptors. These ionotropic receptors are ion channels that are permeable to cations (i.e. sodium [Na+] and calcium [Ca2+]), which in turn depolarize the neuron and/or promote intracellular signaling cascades. There are eight G-protein-coupled metabotropic glutamate receptors subtypes (mGluR1-8) that are divided into three distinct groups that are based on their homology and function: Group I (mGluR1 and mGluR5), Group II (mGluR2 and mGluR3), and Group III (mGluR4, mGluR6, mGluR7, and mGluR8). Group I mGluRs are localized on the postsynaptic neuron and are coupled to G**_q_**/G**_11_** subunits; whereas Group II and Group III are localized on the presynaptic neuron and are couple to G**_i_**/G**_o_** subunits (Auer et al., 2000; Gartlehner et al., 2016; Autry et al., 2011). mGluRs can mediate intracellular signaling cascades by activating second messenger pathways and/or through its βγ subunits. Group I and Group II mGluRs have been investigated in the pathophysiology and treatment of MDD. Specifically, mGluR5 (e.g. AZD2066 and RO4917523) and mGluR2/3 (RO4995819) negative modulators have been tested in Phase II clinical trials for treatment-resistant patients, and some compounds (e.g. RO4917523and RO4995819) have shown promising results (Mcintyre et al., 2017; Beneyto et al., 2007; Beneyto and Meador-Woodruff, 2008; Azbill et al., 2000). Assessing all glutamate receptors and their respective implications in MDD are too wide and beyond the scope of this review. Therefore, this present review will primarily focus on NMDA receptors.

Although majority of the clinically available antidepressant drug classes work to produce an immediate increase in the monoaminergic neurotransmitter concentrations, there is still a population of patients that do not respond to these medications. This lends further support for the revised monoaminergic theory which states that depleted monoaminergic neurotransmitters concentrations or functions may play more of a neuromodulatory role to other neurobiological neurotransmission systems in the central nervous system, rather than a major direct role in MDD (Berman et al., 2002; Mcintyre et al., 2017; Yamakura and Shimoji, 1999; Zarate et al., 2013). Thus, more recent research has focused on finding novel, non-monoaminergic based, receptor targets for treatment-resistant depression. In particular, the glutamatergic system has become a focal point for drug development research.

Attempts to develop antidepressants that work on other neurotransmitter systems are currently ongoing. One of such neurotransmitter system is the excitatory glutamatergic neurotransmitter pathway that appears to be important in the pathophysiology of depression disorders. Clinical research has used both indirect and direct measures to evaluate the glutamatergic system in patients suffering from MDD, and have found evidence of glutamatergic dysfunction in MDD. For example, clinical studies that have used indirect measures for analysis, such as plasma, cerebrospinal fluid, and serum concentrations, have found differences in glutamate and glutamine in patients diagnosed with MDD as compared to healthy controls. Specifically, several studies have found increased concentrations of glutamate in plasma and increased concentration of glutamine in the cerebrospinal fluid of MDD patients. Furthermore, chronic antidepressant drug treatment has been found to reduce the serum and plasma glutamate concentrations, as well as cerebrospinal fluid glutamine concentrations. Also, antidepressants are known to impact glutamatergic neurotransmission in a variety of ways; for example, chronic antidepressant use is associated with reduction of glutamatergic neurotransmission processes, including a reduction in the presynaptic release of glutamate in the hippocampus and cortical areas. Similarly, the chronic administration of antidepressants significantly reduces depolarization-evoked release of glutamate in experimental animal models. Stress is known to enhance the release of glutamate in experimental animal models, and antidepressants inhibit stress-induced presynaptic release of glutamate in these models (Berman et al., 2002; Mcintyre et al., 2017; Yamakura and Shimoji, 1999; Zarate et al., 2013). These findings suggest that these monoaminergic systems selective-acting antidepressant drugs are neuromodulating the functions of the glutamatergic neurotransmission system. In addition, postmortem studies have revealed significant increase in the frontal and dorsolateral prefrontal cortex of depressed patients. Likewise, structural neuroimaging studies have consistently found volumetric changes in the brain areas of depressed patients in which glutamatergic neurons and their connections are most abundant, including the amygdala and hippocampus (Yamakura and Shimoji, 1999; Zarate et al., 2013).

Fig. 1b showed a schematic representation of the NMDA-glutamatergic receptor (NMDAR) heteromeric complex. The NMDA-glutamatergic receptor (NMDAR) is activated when the endogenous co-agonist neurotransmitters- glutamate (or d-aspartate) and glycine (or d-serine) bind to it. When activated, NMDAR allows non-selective positively charged ions (cations) such as Ca**^2+^**, Na**^+^** and K**^+^** to flow through the cell membrane. The NMDA receptor is very important for controlling synaptic plasticity, learning and memory. While the opening and closing of the ion channel is primarily gated by ligand binding, the current flow through the ion channel is voltage dependent. Extracellular magnesium (Mg**^2+^**) and zinc (Zn**^2+^**) ions can bind to specific sites on the receptor, blocking the passage of other cations through the open ion channel. Depolarization of the cell dislodges and repels the Mg**^2+^** and Zn**^2+^** ions from the pore, thus allowing a voltage-dependent flow of sodium (Na**^+^**) and small amounts of calcium (Ca**^2+^**) ions into the cell and potassium (K**^+^**) out of the cell. Currently, the NMDA-glutamatergic receptor (NMDAR) is a heteromeric complex that has three (3) different subunits with a total of fourteen (14) isoform variants for all of these subunits. The NMDA receptor heteromeric complex interacts with multiple intracellular proteins by these three different subunits namely: GluN1 (NR1), GluN2 (NR2) and GluN3 (NR3). The GluN1 subunits have eight different isoform variants generated by alternative splicing from a single gene GRIN1. These different isoform variants of GluN1 subunits are GluN1-1a (the most abundantly expressed isoform variant), GluN1-1b, GluN1-2a, GluN1-2b, GluN1-3a, GluN1-3b, GluN1-4a and GluN1-4b. In vertebrates, there are expressions of four different isoform variants of GluN2 subunits which are GluN2A, GluN2B, GluN2C and GluN2D that are encoded by the GRIN2A, GRIN2B, GRIN2C and GRIN2D genes respectively. Glutamate binding site and the control of the Mg**^2+^** block are formed by the GluN2B subunit isoform variant. Furthermore, GluN2B is predominant in the early postnatal brain, but the number of GluN2A subunits grows, and eventually GluN2A subunits outnumber GluN2B. This is called the GluN2B-to-GluN2A developmental switch, and is notable because of the different kinetics each GluN2 subunit isoform variant lends to the NMDA receptor. For instance, greater ratios of the GluN2B subunit leads to NMDA receptors which remain open longer compared to those with more GluN2A. Unlike GluN1 subunits, the GluN2 subunits are expressed differentially across various cell types and control the electrophysiological properties of the NMDA receptor. The GluN2B subunit isoform variant is mainly present in immature neurons and in extrasynaptic locations. The basic structure and functions associated with the NMDA receptor can be predominantly attributed to the GluN2B subunit. The GluN2B subunit has been involved in modulating activity such as learning, memory, processing and feeding behaviors, as well as being implicated in number of human pathological derangements such as MDD. Late in the 20th century, the GluN3 subunits were discovered with two isoform variants GluN3A and GluN3B that are encoded by the GRIN3A and GRIN3B genes respectively. Furthermore, the family of GluN3 subunits (i.e, GluN3A and GluN3B isoform variants) also possesses a glycine binding site each that exhibit an inhibitory (antagonistic/negative modulatory) effect on NMDA receptor activity/function in contrast to the stimulatory (agonistic/positive modulatory) effect exhibited by the GluN1 subunits when they are bound to the co-agonist glycine. This depicts that the co-agonist glycine binds to any of the GluN3 subunit isoform variants to inhibit and antagonize (negative modulation) the activation of NMDA receptor activity/function. Following the studies carried out by Das in 1998 demonstrating the existence of these two (2) varieties of the GluN3 subunits (GluN3A and GluN3B), which are coded by different genes. The GluN3A variant is expressed throughout the CNS, but expression of the GluN3B variant is restricted to motor neurons. Unlike the GluN2 subunit, GluN3 is a regulatory subunit and its presence decreases the ionic currents generated by activation of the GluN1/GluN2 heteromers. Further studies also showed that the co-expression of GluN1/GluN3B heteromers form excitatory glycine receptors that are insensitive to glutamate/D-aspartate/NMDA and Mg^2+^ blockade. Based on this evidence, it has been postulated that these receptors may be involved in the activation of silent NMDA-alone synapses. All the NMDAR subunits share a common membrane topology that is dominated by a large extracellular N-terminus, a membrane region comprising three transmembrane segments, a re-entrant pore loop, an extracellular loop between the transmembrane segments that are structurally not well known, and an intracellular C-terminus, which are different in size depending on the subunit and provide multiple sites of interaction with many intracellular proteins. Multiple receptor isoform variants with distinct brain distributions and functional properties arise by selective splicing of the GluN1 transcripts and differential expression of the GluN2 subunits. The glycine-binding site modules of the GluN1 and GluN3 subunits and the glutamate-binding site module of the GluN2A subunit have been expressed as soluble proteins, and their three-dimensional structure has been revealed at atomic resolution by x-ray crystallography. The GluN1—GluN2 dimer is therefore considered to be the basic functional organisation structure in each receptor. It contains various sites for the binding and recognition of different ligands, which may be either physiological or pharmacological. In this way, each ionotropic receptor subunit has a very similar molecular structure, divided into 4 functional domains. These consist of an amino-terminal extracellular domain (NTD); a ligand-binding domain (LBD); a transmembrane region formed by four hydrophobic segments (M1 to M4), with M2 partially entering the membrane to form the ion channel; and a carboxyl tail domain (CTD) in the intracellular region. In addition to natural glycine and glutamate binding sites in the GluN1—GluN2 dimer, the extracellular region of GluN2 in particular contains binding sites for endogenous ligands such as polyamines, which are redox sites for protons and zinc. They may exert a regulatory effect on NMDA receptor activity by permitting increases or decreases in calcium flux through the receptor under physiological and/or pathological conditions. At the same time, exogenous ligands for steroids, ethanol, and ifenprodil, and a few synthetic molecules, act as experimental tools for the study of NMDA receptor properties and aid in the development of therapeutically useful antagonists. Homomers of the GluN2 subunit do not generate functional receptors, and are only considered as modulators. Homomers of GluN1 subunits produce channels that are activated by glutamate, aspartate or NMDA in the presence of glycine (or d-serine), but they produce very low amplitude currents compared to receptors formed by GluN1—GluN2 combined (Berman et al., 2002; Mcintyre et al., 2017; Yamakura and Shimoji, 1999; Zarate et al., 2013; Yamakura et al., 1993).

A functional NMDA-glutamatergic receptor must comprise of a minimum heterotetramer complex with at least two obligatory GluN1 subunits and two regionally localized variable GluN2 subunits. The GluN1/GluN2B transmembrane segments are considered to be the part of the receptor that forms the binding pockets for uncompetitive NMDA receptor antagonists. The high affinity sites for glycine antagonist/inverse agonist/partial agonist are also exclusively displayed by the GluN1/GluN2B subunits of NMDA receptor. It is claimed that the presence of three (3) binding sites within the receptor namely, A644 on the GluN2B subunit with A645 and N616 on the GluN1 subunit, are important for binding of ketamine, memantine and other uncompetitive NMDA receptor antagonists (Berman et al., 2002; Yamakura and Shimoji, 1999; Zarate et al., 2013; Yamakura et al., 1993). As earlier mentioned, unlike other ligand-gated ion channels; NMDA-glutamatergic receptors require two distinct mechanisms in order to be activated. First, NMDA-glutamatergic receptor channels require co-agonist binding at the glycine (or d-serine) binding site on the GluN1 subunit and at the glutamate (or d-aspartate) binding site on the GluN2 subunit. Thus, if one of these co-agonists (glycine/D-serine or glutamate/D-aspartate) is not bound to their respective binding site, the ion channel will not open. Second, the NMDA-glutamatergic receptor channels are blocked by magnesium (Mg**^2+^**) ions during the resting state. Depolarization of the neuron is required to dispel the Mg**^2+^** ion from NMDA-glutamatergic receptor channels, which is usually achieved by activation of AMPA receptor-mediated depolarization of the postsynaptic membrane, which relieves the voltage-dependent channel block by Mg**^2+^**. The NMDA-glutamatergic receptor ion channel is non-selective and will allow both sodium (Na**^+^**) and calcium (Ca**^2+^**) ions to enter and potassium (K**^+^**) ions out of the cell. The influx of Ca**^2+^** is associated with the induction of various signaling cascades (Berman et al., 2002; Yamakura and Shimoji, 1999; Zarate et al., 2013; Yamakura et al., 1993).

Several postmortem studies have also found changes in the expression of NMDA-glutamatergic receptor subunits in MDD patients, which are likely compensatory effects to the changes in glutamatergic substrate concentrations, and appear to be brain region specific. For example, the GluN2B and GluN2C subunits have been shown to have increased expression in the locus coeruleus in postmortem tissue of MDD patients. Additionally, the expression of GluN2A subunits has been found to be elevated in the lateral amygdala. Furthermore, MDD patients have shown an increase in glutamate binding in the hippocampus and a greater sensitivity to glutamate as measured by intracellular calcium influx. On the other hand, the GluN2A and GluN2B subunits transcription have been shown to be reduced in the perirhinal and prefrontal cortices in postmortem tissue from MDD patients. Moreover, postmortem studies have found decreased levels of the GluN1subunit in the superior temporal cortex and prefrontal cortex. The GluN1 and GluN2 subunits are required for functional NMDA-glutamatergic receptor heteromeric complexes, and thus, increase/decrease in the levels of these GluN1/GluN2 subunits can be interpreted as changes in total number of functional NMDA-glutamatergic receptors. Based on these previous experimental results, it was hypothesized that depression is associated with the hyperfunction of NMDA-glutamatergic receptors in subcortical regions (i.e. hippocampus, locus coeruleus, and amygdala); whereas at the same time, depression is associated with the hypofunction of NMDA-glutamatergic receptors in cortical regions (i.e. prefrontal, perirhinal and temporal cortices). And this finding has led to a conclusion that postulates the new “Glutamatergic hypothesis of depression” which is now moving our understanding of the pathophysiology of MDD a step further from the several decades’ old “Monoaminergic theory of depression” (Berman et al., 2002; Yamakura and Shimoji, 1999; Zarate et al., 2013). Collectively, clinical data suggest the involvement of the glutamatergic system in the pathophysiology of MDD or bipolar depression or schizoaffective depression, which includes disruptions in glutamatergic substrate concentrations and NMDA-glutamatergic receptor alterations (Yamakura and Shimoji, 1999; Zarate et al., 2013; Yamakura et al., 1993). Although the role of glutamatergic systems is yet to be fully elucidated, but a “proof of concept” clinical study reported that the non-competitive NMDA-glutamatergic receptor antagonist ketamine produced rapid-onset and prolonged antidepressant effects in patients suffering from MDD or bipolar depression or schizoaffective depression (Zarate et al., 2013). Ketamine is a potent, high-affinity, noncompetitive N-methyl-d-aspartate (NMDA) receptor antagonist that has long been used in anesthesia and is a common drug of abuse in some parts of the world. A number of preclinical and clinical studies have demonstrated rapid antidepressant effects of ketamine. Multiple studies have suggested that a single dose of intravenous ketamine at sub-anaesthetic doses produces rapid relief of depression, even in treatment-resistant patients, that may persist for 1 week or longer. Unfortunately, ketamine is associated with neurocognitive dysfunction, dissociative, and psychotomimetic properties that make it unsuitable as a long-term treatment for depression. Still, this has generated tremendous interest in developing new drugs that will target the glutamatergic neurotransmission mechanisms for the treatment of MDD or bipolar depression or schizoaffective depression. These potential drug targets are the NMDA-glutamatergic receptor as antagonist or inverse agonist or partial agonist; metabotropic glutamatergic receptors as positive or negative modulator; excitatory amino acid transporter-2 (EAAT-2) as a reuptake enhancer;and as a terminal presynaptic glutamate release inhibitor (Yamakura and Shimoji, 1999; Zarate et al., 2013; Yamakura et al., 1993). Finally, the structure of mGluRs consists of a protein chain that crosses the membrane seven times. To date, the eight units named mGluR1 through mGluR8 that have been cloned, are classified according to the following: (a) the homology of their amino acids (70% homology among members of the same class, and 45% homology between different classes); (b) in response to their agonists, and (c) the signal paths for second messengers. These previously mentioned Ionotropic receptors are categorised according to whether their specific agonists have an affinity for N-methyl-d-aspartate (NMDA), α-amino-3-hydroxy-5-methyl-4-isoxazole (AMPA), or kainic acid (KA). Ionotropic receptors are heteromers constituted by different subunits, which give the receptors different physiological and pharmacological properties. The AMPA receptors are structured as combinations of GluA1 (GluR1), GluA2 (GluR2), GluA3 (GluR3), and/or GluA4 (GluR4) subunits which form an ion channel permeable to Na+. However, it has been shown that AMPA receptors whose structure does not include a GluA2 subunit are highly permeable to calcium (Ca^2+^) ions. This is due to the presence of a residue of arginine (R), an amino acid present in position R586 in the TMII region of GluA2. In contrast, subunits GluA1, GluA3 and GluA4 present a glutamine (Q) residue at position Q582 of the GluA1 subunit protein. The Kainate receptors are protein heteromers formed by combinations of the GluK1 (GluR5), GluK2 (GluR6), and/or GluK3 (GluR7) subunits, together with GluK4 (KA1) and/or GluK5 (KA2) subunits. The combination of GluK5 and GluK1 forms a functional receptor that is permeable to calcium (Ca^2+^) ions (Yamakura and Shimoji, 1999; Zarate et al., 2013; Yamakura et al., 1993).

### 4.1 N-methyl-d-aspartate (NMDA)-glutamatergic ionoceptor blockers

The NMDA-glutamatergic ionoceptor blockers are group of drug substances that exhibit either pure antagonist or inverse agonist or partial agonist (mixed agonist-antagonist) pharmacological properties at the NMDA receptors. Their pharmacological mechanism of actions can either be through a direct blockade of the NMDA receptors (such as rapastinel, apimostinel and ketamine) or via an indirect blockade of the NMDA receptors (such as riluzole) (Yamakura and Shimoji, 1999; Zarate et al., 2013; Yamakura et al., 1993).

#### 4.1.1 Selective antagonist or inverse agonist or partial agonist at the GluN1 subunit glycine binding-site of NMDA receptor [Direct-acting GluN1 subunit-selective NMDA-glutamatergic receptor antagonist/inverse agonist/partial agonist]

Rapastinel (former developmental code names GLYX-13, BV-102) is a novel antidepressant that is under development by Allergan (previously Naurex) as an adjunctive therapy for the treatment of treatment-resistant major depressive disorder. It is a centrally active, intravenously administered (non-orally active) amidated tetrapeptide (Thr-Pro-Pro-Thr-NH**_2_**) that acts as a selective, weak partial agonist (mixed antagonist/agonist) of an allosteric site of the glycine site of the NMDA receptor complex (Emax ≈ 25%). The drug is a rapid-acting and long-lasting antidepressant as well as robust cognitive enhancer by virtue of its ability to both inhibit and enhance NMDA receptor-mediated signal transduction. The novel compound, GLYX-13 (rapastinel), which is a tetrapeptide (TPPT-amide), was developed for the treatment of MDD with the goal of producing rapid-onset antidepressant effects without producing psychotomimetic side effects (Yamakura and Shimoji, 1999; Zarate et al., 2013; Yamakura et al., 1993). Unlike the GluN2B subunit selective NMDA receptor antagonists and the channel blockers (GluN2 subunit unselective NMDA receptor antagonists), GLYX-13 (rapastinel) binds selectively to the GluN1 subunit glycine-binding site of the NMDA receptor and acts as a functional partial agonist with this difference in pharmacological action believed to reduce psychotomimetic side effects. Typically, partial agonists will produce agonistic effects at low doses or in the absence of the receptor's site full agonist (glycine), but will produce antagonistic effects at high doses or in the presence of the receptor's site full agonist (glycine). In a Phase II clinical study comprising of 112 MDD patients, GLYX-13 (rapastinel) produced rapid and sustained antidepressant effects following a single infusion (5.0–10.0 mg/kg; 3–15 min infusion), and, most importantly, did not produce psychotomimetic effects. Specifically, the antidepressant effects of GLYX-13 (rapastinel) were apparent at the end of day one and persisted until day seven following the single infusion. A Phase II double-blind, placebo control, multi-dose clinical trial has also been done (NCT01684163). GLYX-13 (rapastinel) and its congener compounds do not bind directly to the glycine binding site of the GluN1 subunit of NMDA receptor but rather bind to a different regulatory allosteric site on the GluN1 subunit of NMDA receptor complex that serves to allosterically modulate the glycine binding site. As such, rapastinel is technically an allosteric modulator of the glycine site of the NMDA receptor, and hence is more accurately described as a functional glycine site weak partial agonist. In addition to its antidepressant effects, rapastinel has been shown to enhance memory and learning in both young adult and learning-impaired, aging rat models. It has been shown to increase Schaffer collateral-CA1 long-term potentiation in vitro. In concert with a learning task, rapastinel has also been shown to elevate gene expression of hippocampal GluN1, a subunit of the NMDA receptor, in three-month-old rats. Neuroprotective effects have also been demonstrated in Mongolian Gerbils by delaying the death of CA1, CA3, and dentate gyrus pyramidal neurons under glucose and oxygen-deprived conditions. Additionally, rapastinel has demonstrated antinociceptive activity, which is of particular interest, as both competitive and noncompetitive NMDA receptor antagonists are ataxic at analgesic doses, while rapastinel and other glycine subunit ligands are able to elicit analgesia at non-ataxic doses (Mcintyre et al., 2017; Gartlehner et al., 2016; Yamakura and Shimoji, 1999; Zarate et al., 2013; Yamakura et al., 1993).

In addition to GLYX-13 (rapastinel), another novel congener compound NRX-1074 (Apimostinel) has been developed, which is similar to GLYX-13 (rapastinel) pharmacologically; however, NRX-1074 (Apimostinel) is an orally bioavailable compound and is more potent than GLYX-13 (rapastinel). In a 2014 Phase I clinical trials, NRX-1074 (Apimostinel) was well tolerated. As of 2015, an intravenous formulation of apimostinel is in a phase II clinical trial for MDD, and an oral formulation is concurrently in phase I trials for MDD. Like rapastinel, It is under development as an adjunctive therapy for treatment-resistant depression. Furthermore on NRX-1074 (Apimostinel), clinical trial recruitment for Phase I safety and pharmacokinetic study (NCT01856556) and Phase II multi-dose single infusion for patients with MDD (NCT02067793) has been done. However, apimostinel is 100-fold more potent by weight and orally stable, whereas rapastinel must be administered via intravenous injection, is orally-active. Apimostinel is intended by Allergan as an improved, follow-up drug to rapastinel. Similarly to rapastinel, apimostinel is an amidated tetrapeptide, and has almost an identical chemical structure to rapastinel, but has been structurally modified via the addition of a benzyl group. The drug has shown rapid antidepressant effects in pre-clinical models of depression. In addition, similarly to rapastinel, it is well-tolerated and lacks the schizophrenia-like psychotomimetic effects of other NMDA receptor antagonists such as ketamine (Mcintyre et al., 2017; Gartlehner et al., 2016; Yamakura and Shimoji, 1999; Zarate et al., 2013; Yamakura et al., 1993).

#### 4.1.2 Unselective antagonist or inverse agonist or partial agonist at the GluN2 subunits glutamate binding-site of NMDA receptor [Direct-acting GluN2 subunits-unselective NMDA-glutamatergic receptor antagonist/inverse agonist/partial agonist]

Ketamine is a non-competitive and unselective antagonist for the GluN2 subunits of NMDA-glutamatergic receptor (aka channel blocker) that binds to the phencyclidine binding site inside the ion channel of the NMDA receptor, blocking the channel in a way that is similar to how Mg2+ ion blocks NMDA receptors, and is unselective for the GluN2A-D subunits of the NMDA receptor channel. Non-competitive NMDA-glutamatergic ionoceptor antagonists that exhibit a direct action on the excitatory glutamatergic neurotransmission system such as ketamine are now being promoted for off-label use in the treatment of MDD or bipolar depression or schizoaffective depression. Sub-anaesthetic low dose ketamine has been found to possess a rapid-onset antidepressant action with a minimal dissociative anaesthetic effect clinically. Because of this property clinical psychiatrists are now using this drug as an adjunct or augmenting pharmacotherapeutic agent in the management of major depressive disorder or bipolar depression or schizoaffective depression so as to facilitate and enhance fast clinical remission. The indication of ketamine for this purpose in MDD or bipolar depression or schizoaffective depression has not been officially approved by the FDA. Ketamine is a potent, high-affinity, non-competitive N-methyl-d-aspartate (NMDA) receptor antagonist that has long been used in anaesthesia and is a common drug of abuse in some parts of the world. A number of preclinical and clinical studies have demonstrated rapid antidepressant effects of ketamine. Multiple studies have suggested that a single dose of intravenous ketamine at sub-anaesthetic doses produces rapid relief of depression, even in treatment-resistant patients, that may persist for 1 week or longer. Unfortunately, ketamine is associated with neurocognitive dysfunction, dissociative, and psychotomimetic properties that make it unsuitable as a long-term treatment for depression. Still, a number of other NMDA-glutamatergic receptor antagonist or inverse agonist or partial agonist; metabotropic glutamatergic receptors positive or negative modulator; excitatory amino acid transporter-2 (EAAT2) reuptake pump enhancer; and terminal presynaptic glutamate release inhibitor are under investigation as potential antidepressants for clinical use (Mcintyre et al., 2017; Gartlehner et al., 2016; Yamakura and Shimoji, 1999; Zarate et al., 2013; Yamakura et al., 1993).

In the Berman et al. (2000) study, the non-competitive NMDA-glutamatergic receptor antagonist ketamine was first used in a “proof of concept” randomized double-blind study to assess the effects of ketamine on MDD in seven patients who received both vehicle (placebo) and ketamine treatment (counter-balanced). A single, sub-anaesthetic dose of ketamine (0.5 mg/kg) was intravenously (i.v.) infused over 40 min, and the antidepressant effects of ketamine were assessed using the Hamilton Depression Rating Scale (HDRS) and Beck Depression Inventor (BDI). In comparison, an anaesthetic dose for ketamine in humans ranges from 1.0 mg/kg to 4.5 mg/kg intravenous and from 6.5 mg/kg to 13.0 mg/kg intramuscular. In this study, ketamine produced rapid, within four hours, and prolonged antidepressant effects that lasted up to 72 h as compared to placebo control. This rapid antidepressant effect of ketamine is far superior to the 4–12 week delay with current antidepressant drugs. The hallucinogenic (or psychotomimetic) effects (e.g. out of body experience, hallucinations, etc.) of ketamine subsided (within two hours) prior to the onset of the antidepressant effects as measured by the Visual Analog Scales for intoxication “high” (VAS-high) and Brief Psychiatric Rating Scale (BPRS). This was the first clinical study to demonstrate that glutamatergic drugs may be effective for the treatment of MDD.

In another clinical study conducted by Zarate and colleagues to assess the antidepressant effects of ketamine in patients with treatment-resistant MDD and to determine a better understanding of the duration of the antidepressant effects; following a single low-dose 0.5 mg/kg infusion of ketamine, treatment-resistant patients showed a significant reduction in depression scores at 110 min that lasted up to seven days as measured by HDRS. Specifically, 71% of the patients achieved response criteria one day after the infusion, while 29% achieved full remission. Additionally, 35% maintained response criteria on day seven. Again, the hallucinogenic (or psychotomimetic) effects diminished before the onset of the antidepressant effects of ketamine (within two hours). This study confirmed the finding in the Berman et al. (2000) study that ketamine produces rapid and prolonged antidepressant effects in the treatment of depression and extended ketamine's efficacy to treatment-resistant MDD.

Another study conducted by Ghasemi et al. (2014) to compared the effects of ketamine and electroconvulsive therapy (ECT) in patients suffering from MDD. This study found out that both ketamine and ECT produced antidepressant effects; however, ketamine produced superior antidepressant effects in terms of fast response onset. For example, ketamine produced rapid antidepressant effects starting at 24 h; whereas, the antidepressant effects of ECT were not expressed until after 48 h. The antidepressant effects of both ketamine and ECT lasted until the completion of the study, which was seven days. These results suggest that ketamine is as efficacious, if not more efficacious, as ECT for treating MDD.

In addition to these previously mentioned studies, several other clinical studies conducted by Lara et al. (2013); Lapidus et al. (2014) and Zigman and Blier, 2013 have also found out that low-dose ketamine produce rapid and sustained antidepressant effects in patients with MDD; a rapid reduction in suicidal ideation but produced some neurocognitive dysfunction in patients with treatment-resistant MDD.

An anti-anhedonic effect of ketamine treatment in treatment-resistant bipolar depression was recently demonstrated by Lally et al., 2014. In a randomized, placeb-controlled, double-blind crossover design, 36 treatment-resistant bipolar depression patients were treated with a single, low intravenous dose of 0.5 mg/kg ketamine. They found that ketamine rapidly reduced anhedonia in these patients within 40 min and that these effects preceded reductions in other depressive symptoms. Also, the decrease in anhedonic symptoms persisted up to 14 days. The authors concluded that these findings demonstrate the importance of glutamatergic mechanisms for the treatment of treatment-refractory bipolar depression and especially for the treatment of anhedonia symptoms. Currently, preclinical research is evaluating the pharmacological and intracellular effects that are responsible for the antidepressant effects of ketamine, which will aid the development of novel glutamatergic antidepressant drugs (Mcintyre et al., 2017; Gartlehner et al., 2016; Yamakura and Shimoji, 1999; Zarate et al., 2013; Yamakura et al., 1993). The postulations from the studies done by Panos et al., 2016 and Wray et al., 2018 are new insights into the other possible mechanism of action for NMDA antagonist such as ketamine but these postulations are yet to be universally accepted. In addition, the postulation from the studies by these two groups of researchers are conflicting and contradictory to each other. Panos et al., 2016 postulated that negative allosteric modulation or selective inhibition of NMDARs localized on GABAergic interneurons with GABA-A receptors containing alpha 5 subunits (alpha 5 GABA-NAMs) in the prefrontal cortex (restricted brain localization) mediate the rapid antidepressant-like actions of ketamine, perhaps via an AMPA receptor-dependent increase in coherent neuronal circuit activity. While Wray et al., 2018 hypothesized that ketamine would translocate Gα from lipid rafts to non-raft microdomains, similarly to other antidepressants but with a distinct, rapid/fast onset treatment duration of action. Other NMDA antagonists did not translocate Gα from lipid raft to non-raft domains. The ketamine-induced Gα plasma membrane redistribution allows increased functional coupling of Gα and adenylyl cyclase to increase intracellular cyclic adenosine monophosphate (cAMP). Moreover, increased intracellular cAMP increased phosphorylation of cAMP response element-binding protein (CREB), which, in turn, increased BDNF expression. The ketamine-induced increase in intracellular cAMP persisted after knocking out the NMDA receptor indicating an NMDA receptor independent effect. Furthermore, the ketamine metabolite (2R,6R) hydroxynorketamine (HNK) also induced Gα redistribution and increased cAMP. These results reveal a novel antidepressant mechanism mediated by acute ketamine treatment that may contribute to ketamine’s powerful antidepressant effect. They also suggest that the translocation of Gα from lipid rafts is a reliable hallmark of antidepressant action that might be exploited for diagnosis or drug development.

#### 4.1.3 selective antagonist or inverse agonist or partial agonist at the GluN2B subunit glutamate binding-site of NMDA receptor [Direct-acting GluN2B subunit-selective NMDA-glutamatergic receptor antagonist/inverse agonist/partial agonist]

The Pfizer pharmaceutical company developed the potent GluN2B subunit selective NMDA receptor antagonist CP-101,606 (traxoprodil) as a neuroprotectant for head injury and stroke, but later it was evaluated as an adjunctive treatment for patients with treatment-resistant MDD. The selectivity of traxoprodil for GluN2B subunits of the NMDA receptor complex was believed to reduce the psychotomimetic effects that have been associated with the nonspecific NMDA receptor antagonist ketamine. A single eight hour infusion of traxoprodil (0.75 mg/kg per hour for 1.5 h, then 0.05 mg/kg per hour for 6.5 h) was evaluated as an adjunctive treatment to paroxetine (40.0 mg/day) in a double-blind between subjects design clinical study. Traxoprodil produced rapid (five days) antidepressant effects with 60% of the patients meeting response criteria. However, traxoprodil produced psychotomimetic effects in four of the nine patients that met response criteria. Although a phase II clinical trial was conducted in 2005–2006 to evaluate the effects of monotherapy traxoprodil in patients with treatment-resistant depression (NCT00163059), to date, there are no published results from these clinical trials (Mcintyre et al., 2017; Gartlehner et al., 2016; Zarate et al., 2013).

Recently, another GluN2B subunit selective NMDA receptor antagonist MK-0657, that developed for the treatment of Parkinson's disease, was the first oral formulation of NMDA receptor antagonist to be tested in treatment-resistant MDD patients. This was a double-blind, placebo-controlled study in which the patients received either MK-0657 (4.0–8.0 mg/d) or placebo for 12 days. MK-0657 produced inconsistent antidepressant effects from day 5 to day 12 as measured by both Hamilton Depression Rating Scale (HDRS) and Beck Depression Inventor (BDI). Furthermore, MK-0657 failed to produce a significant reduction in depression symptoms as measured by Montgomery-Asberg Depression Rating Scale (MADRS). MK-0657 did not produce psychotomimetic or adverse side effects. One possible explanation for the inconsistent results is that the study was terminated after only five patients completed both phases of the study. Early termination of the study was due to recruitment challenges (Mcintyre et al., 2017; Gartlehner et al., 2016; Zarate et al., 2013).

#### 4.1.4 Excitatory amino acid transporter-2 (EAAT-2) reuptake enhancer and terminal presynaptic glutamate release inhibitor [Indirect-acting unselective glutamatergic receptors antagonist]

The EAAT-2 glutamate reuptake enhancer and terminal presynaptic glutamate release inhibitor- Riluzole, which is approved by the FDA for the treatment of amyotrophic lateral sclerosis (ALS) has been evaluated under a number of conditions for the treatment of MDD including monotherapy, adjunctive therapy, and ketamine relapse prevention. Because of its unique mechanism of action; Riluzole is being referred to as an Indirect-acting unselective glutamatergic receptors antagonist due to the fact that its spectrum of pharmacological action extends to affect both the ionotropic (NMDA, AMPA and kainate) glutamatergic receptors and the metabotropic (mGluR1-8) glutamatergic receptors. Riluzole was evaluated as a treatment for MDD because of its dual pharmacological effects on the glutamatergic system. Specifically, riluzole increases the reuptake of glutamate into astrocytes via EAAT-2 and also inhibits terminal presynaptic glutamate release, which produces pharmacological actions similar to the effects of the NMDA receptor antagonists such that riluzole can reduce NMDA receptor activation by decreasing the synaptic concentrations of glutamate available to bind to postsynaptic NMDA receptors. The antidepressant effects of riluzole were first evaluated in an open-label clinical study in patients with treatment-resistant MDD. In the open-label clinical study, daily riluzole (mean dose of 169 mg/day) produced antidepressant effects on weeks three through week six as compared to baseline MADRS score. There was not a placebo control in this study by Zarate et al. (2004). In another small scale clinical study (n = 10), adjunctive riluzole (100 mg/day) treatment produced a rapid decrease in depressive symptoms from week one through week six as compared to baseline HDRS scores. There was no placebo control group in this study by Sanacora et al. (2007). Two double-blind clinical studies evaluated riluzole as relapse prevention for patients that response to a single infusion of ketamine; however, both studies found that riluzole was no more effective than placebo for ketamine relapse prevention. Moreover, riluzole did not produce antidepressant effects in patients that did not response to ketamine infusions (i.e. ketamine non-responders). In general, riluzole was well tolerated in these studies and psychotomimetic effects were not observed. At the time of this review, two Phase II double-blind, placebo control, adjunctive treatment clinical trial are underway for patients with treatment-resistant MDD (NCT01204918 and NCT01703039) (Sanacora et al., 2007; Mcintyre et al., 2017; Gartlehner et al., 2016; Zarate et al., 2013).

### 4.2 New selective monoamine oxidase inhibitors (MAOIs)

Monoamine oxidase inhibitors (MAOIs) are a class of drugs that inhibit the activity of one or both monoamine oxidase enzymes namely: monoamine oxidase A (MAO-A) and monoamine oxidase B (MAO-B). They have a long history of use as medications prescribed for the treatment of depression. MAOIs act by inhibiting the activity of monoamine oxidase enzyme(s), thus preventing the breakdown of monoamine neurotransmitters and thereby increasing their synaptic availability. There are two isoforms of monoamine oxidase, MAO-A and MAO-B. MAO-A preferentially deaminates serotonin, melatonin, epinephrine, and norepinephrine. MAO-B preferentially deaminates phenethylamine and certain other trace amines; in contrast, MAO-A preferentially deaminates other trace amines, like tyramine, whereas dopamine is equally deaminated by both types. The action of a MAOI is to increase the availability of the monoamine neurotransmitters NE, DA, and 5-HT by blocking their metabolism. They are particularly effective in treating atypical depression, parkinson's disease and several other disorders (Gelenberg et al., 2010; Mcintyre et al., 2017; Gartlehner et al., 2016). The classical MAOIs include both hydrazine and non-hydrazine derivatives. The hydrazine derivatives are phenelzine, nialamide, isocarboxazid, and hydracarbazine while the non-hydrazine derivative is tranylcypromine. These classical MAOIs exhibit unselective and irreversible inhibition, but the newer MAOIs are selective for either MAO-A or MAO-B isoenzyme as well as reversible for MAO-A. Reversible inhibitors of monoamine oxidase A (RIMAs) are a subclass of MAOIs that selectively and reversibly inhibit the activity of MAO-A enzyme. RIMAs are used clinically in the treatment of depression and dysthymia, though they have not gained widespread clinical prescription worldwide. Because of their reversibility and selectivity, RIMAs are safer than the older MAOIs like phenelzine and tranylcypromine. Several selective reversible inhibitors of MAO-A are used outside USA; but only moclobemide is currently approved for use within and outside the United States by the FDA. These selective reversible inhibitors of MAO-A used outside USA are bifemelane (not yet approved by FDA but is available in Japan), pirlindole (not yet approved by FDA but is available in Russia), toloxatone (not yet approved by FDA but is available in France). Furthermore, available selective inhibitors of MAO-B which have been approved by FDA and are currently available for use within and outside USA are selegiline, rasagiline, and safinamide (Reynolds et al., 2011; Kudlow et al., 2012; Rush et al., 2011). The selective MAO-B inhibitor drugs have been approved by the FDA without any dietary restrictions, except in high-dosage treatment, wherein they lose their selectivity. Because of potentially lethal dietary and drug interactions, monoamine oxidase inhibitors have historically been reserved as a last line of treatment, used only when other classes of antidepressant drugs (for example selective serotonin reuptake inhibitors and tricyclic antidepressants) have failed. However, some practitioners have a poor understanding of these potentially lethal dietary and drug interactions with MAOIs; as they can be very serious and life-threatening, concomitant medication use or certain dietary intake (tyramine-containing meals or drinks) must be stringently avoided, monitored, or well overseen as they can cause dangerous or fatal serotonin syndrome or hypertensive crisis (Szegedi et al., 2009; Rush et al., 2011).

### 4.3 Serotonin-norepinephrine reuptake inhibitors (SNRIs)

The new emerging SNRIs include ansofaxine, nefopam and levomilnacipran. Levomilnacipran is the active enantiomer of a racemic SNRI, milnacipran. Milnacipran has been approved for the treatment of fibromyalgia but not depression in the USA and has also been used for the treatment of depression in Europe for many years. In addition to their use in major depression, SNRIs have applications in the treatment of pain disorders including neuropathies and fibromyalgia. SNRIs are also used in the treatment of generalized anxiety disorder, stress urinary incontinence, and vasomotor symptoms of menopause. The pharmacological properties of SNRIs are dose dependent: namely, at low doses they behave essentially like an SSRI; while at medium doses, additional NE reuptake inhibition occurs; and at high to very high doses, they weakly inhibit the reuptake of dopamine with recent evidence showing that the norepinephrine transporter also transports some dopamine as well, since dopamine is inactivated by norepinephrine reuptake pumps in the prefrontal cortex. The prefrontal cortex significantly lack dopamine reuptake transporters (DAT), therefore SNRIs can substantially increase dopaminergic neurotransmission in this part of the brain. Thus, at low doses, the actions of SNRIs are similar to those of the SSRIs, and as the dose increases, the bupropion-like actions progressively kick-in. SNRIs are chemically unrelated to each other. All the SNRIs bind to inhibit the serotonin reuptake (SERT) and norepinephrine reuptake (NET) transporters, as do the TCAs. Furthermore, the SNRIs are all in one drug that combine the pharmacological mechanism of actions for the SSRIs and the NRIs altogether. However, unlike the TCAs, the SNRIs do not have much affinity for other receptors (Mcintyre et al., 2017; Gartlehner et al., 2016). Recently, levomilnacipran, the levorotatory enantiomer of milnacipran, has been found to act as an inhibitor of beta-site amyloid precursor protein cleaving enzyme-1 (BACE-1), which is responsible for β-amyloid plaque formation, and hence may be a potentially useful drug in the treatment of Alzheimer's disease in the near future (Rush et al., 2011). Nefopam is also an analgesic medication asides its SNRI antidepressant activity. It is primarily used to treat moderate to severe, acute or chronic inflammatory pain, neuropathic pain and depression disorders. It is believed to work in the brain and spinal cord to relieve pain. There it is believed to work via unique mechanisms. Firstly it increases the activity of the serotonin, norepinephrine and dopamine, neurotransmitters involved in, among other things, pain signaling. Secondly, it modulates sodium and calcium channels, thereby inhibiting the release of glutamate, a key neurotransmitter involved in pain processing. Nefopam has additional actions in the prevention of shivering (which may be a side effect of other drugs used in surgery) and is being studied as a treatment for desmoid tumors associated with aggressive fibromatosis. Nefopam has been shown to slow or stop desmoid tumors' growth in mice during phase I preclinical trials. Ansofaxine also known as 4-methylbenzoate desvenlafaxine hydrochloride, is a serotonin–norepinephrine–dopamine reuptake inhibitor (SNDRI) which is under development for the treatment of major depressive disorder (MDD). It is described as an SNDRI and prodrug to desvenlafaxine. However, unlike desvenlafaxine, which has invitro IC50 values of 53 nM and 538 nM for inhibition of serotonin and norepinephrine reuptake, respectively, while ansofaxine has invitro IC50 values of 723 nM, 763 nM, and 491 nM for serotonin, norepinephrine, and dopamine reuptake inhibition respectively. As of July 2018, ansofaxine is in preregistration for MDD in the United States, the European Union, Japan, and China. The dopamine reuptake inhibition activity of these drugs may not be of significant benefit/impact in the treatment of primary signs/symptoms caused by depression disorders compared to placebo, but this will definitely be of significant importance/benefit in the treatment of depressed patients with comorbidities such as substance abuse disorders (chronic smokers or chronic alcoholics), hyposexual desire disorder (anorgasmia) due to relative dopamine deficiency, serotonin-induced sexual dysfunction and/or serotonin-induced nocturnal myclonus/akathisia as revealed by recent phase III clinical trials of individuals with treatment-resistant depression using a fixed dose combination of an SSRI or SNRI with lisdexamfetamine (a norepinephrine-dopamine releasing agent) that will mimic and produce the pharmacoactivity of an SNDRI. These occurrences have shed doubt on the potential benefit of dopaminergic augmentation of conventional serotonergic and noradrenergic antidepressant therapy. As such, skepticism has been cast on the promise of the remaining SNDRIs that are still being trialed, such as ansofaxine (currently in phase I trials), in the treatment of depression (Dale et al., 2015; Mcintyre et al., 2017; Gartlehner et al., 2016).

**Fig. 2:**
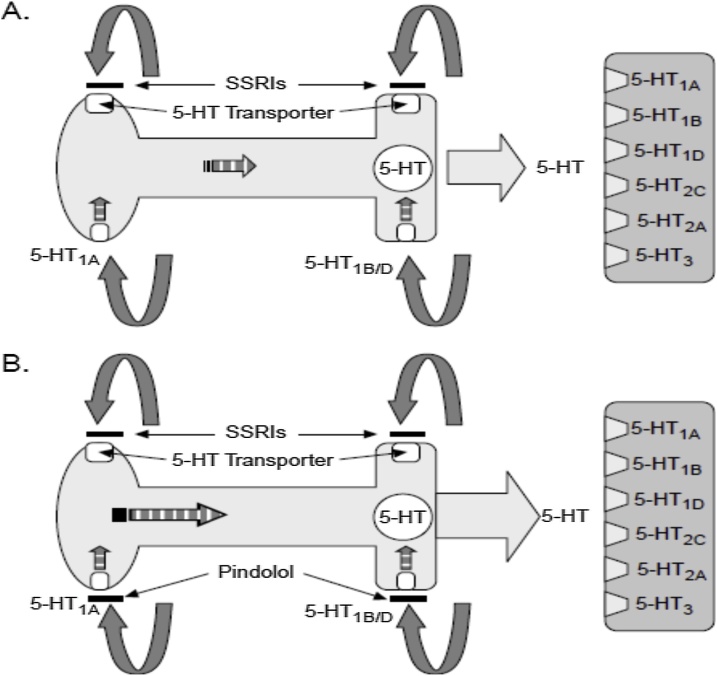
(A) Increase serotonin neurotransmitter at the somatodendritic region and the terminal presynaptic region, which is as a result of an SSRI or SNRI or NASSA or TCA pharmacodynamics effect activates the combinations of the somatodendritic serotonergic 5-HT_1A_/5-HT_7_ autoreceptors and the terminal presynaptic serotonergic 5-HT_1B/1D_ autoreceptors thereby leading to a decrease in the firing rate at the somatodendritic region and a decrease in serotonin neurotransmitter release from the terminal presynaptic region, respectively, thus reducing the antidepressant and the anxiolytic clinical responses (sensitization or pre-desensitization phenomenon/effects). (B) The addition of a drug like a selective 5-HT_7_ autoreceptor antagonist with 5-HT_1A_ autoreceptor partial agonism (such as an atypical antipsychotic); or alternatively a selective 5-HT_1A_ autoreceptor partial agonist (such as buspirone or tandospirone); or alternatively a selective 5-HT_1A_ and 5-HT_1B/1D_ autoreceptors antagonist (such as pindolol) can hasten the antidepressant and the anxiolytic clinical responses to an SSRI or SNRI or NASSA or TCA by bypassing the serotonergic autoreceptors desensitization phenomenon/effects.

The primary mechanism of action for SNRIs is usually explained by their selective inhibition of both the serotonin reuptake transporter (SERT) and norepinephrine reuptake transporter (NET). However, starting with the SERT-inhibition, a more precise mechanism of SNRIs therapeutic action is “delayed disinhibition of serotonergic neurotransmission in at least four key pathways that occur following desensitization of 5-HT_1A_/5-HT_7_ and 5-HT_1B/1D_ autoreceptors”. When an SNRI is administered, it indeed blocks the serotonin reuptake pump, and this happens immediately. However, this action causes a sudden increase in serotonin predominately in the somatodendritic area, and not at the axon terminals where serotonin is presumably needed in order to exert therapeutic actions. Perhaps this explains why SNRIs do not have rapid onset of therapeutic actions. If SNRIs are administered chronically, the sustained increase of serotonin in the somatodendritic area of the serotonergic neurons cause the somatodendritic 5-HT_1A_/5-HT_7_ autoreceptors to desensitize. Once the somatodendritic 5-HT_1A_/5-HT_7_ autoreceptors desensitize, neuronal impulse flow is no longer readily inhibited by serotonin. Thus, neuronal impulse flow is turned on. Another way to say this is that serotonergic neurotransmission is disinhibited, and more serotonin is released from the axon presynaptic terminal. However, this increase is delayed compared with the increase of serotonin in the somatodendritic areas of the serotonergic neurons. This delay is the result of the time it takes for somatodendritic serotonin to desensitize the 5-HT_1A_/5-HT_7_ autoreceptors, and turn on (i.e., disinhibition process) neuronal impulse flow in the serotonergic neurons. As mentioned earlier, this delay may account for why SNRIs do not relieve depression and anxiety immediately. Furthermore, once an SNRI has blocked the serotonin reuptake pumps, increased somatodendritic serotonin concentration desensitized the somatodendritic 5-HT_1A_/5-HT_7_ autoreceptors, disinhibited neuronal impulse flow, and increased release of serotonin from terminal presynaptic membrane, the final step is the desensitization of both the terminal presynaptic 5-HT_1B/1D_ autoreceptors and the postsynaptic serotonin receptors. Desensitization of these receptors inevitably contribute to the therapeutic actions of SNRIs, and/or it could account for the development of tolerance to acute side effects of SNRIs. In summary, the pharmacologic profile of an SNRI is to cause powerful if delayed disinhibition of neurotransmission process in every serotonergic fibre in the central nervous system (CNS). Since different serotonergic pathways are known to mediate different CNS functions, the various therapeutic effects of SNRIs may be mediated by disinhibition in different pathways. Thus disinhibition of serotonergic neurotransmission pathway from midbrain raphe to prefrontal cortex could hypothetically help mediate the antidepressant effects of SSRIs. Similarly, disinhibition of the pathway from midbrain raphe to basal ganglia could hypothetically mediate therapeutic actions of SNRIs in obsessive-compulsive disorder (OCD); while disinhibition of the pathway to mesolimbic cortex and hippocampus, mediate therapeutic actions in panic disorders; and disinhibition of the pathway to hypothalamus, mediate therapeutic actions in bulimia and binge-eating disorder. In each case, SNRI induced disinhibition of serotonergic neurotransmission with delivering of neurotransmitter where it is needed, hypothetically in different places for different psychiatric disorders. Clinical observations support the notion that different pathways mediate the different therapeutic actions of SNRIs, since SNRIs action on different cortical areas depend on which psychiatric disorder is being targeted (Mcintyre et al., 2017; Gartlehner et al., 2016; Kudlow et al., 2012; Rush et al., 2011). Furthermore, Fig. 2 illustrates the detail mechanisms operating in the serotonergic neurotransmission system. The 5-HT_1B/1D_ and 5-HT_1A_/5-HT_7_ autoreceptors play important roles in regulating the terminal presynaptic release of serotonin neurotransmitter and the somatodendritic onset depolarizing activity of serotonergic neurons respectively. It also worth mentioning here that the addition of a drug like a selective 5-HT_7_ autoreceptor antagonist with 5-HT_1A_ autoreceptor partial agonism (such as an atypical antipsychotic); or alternatively a selective 5-HT_1A_ autoreceptor partial agonist (such as buspirone or tandospirone); or alternatively a selective 5-HT_1A_ and 5-HT_1B/1D_ autoreceptors antagonist (such as pindolol) to an SSRI or SNRI or NASSA or TCA treatment decouples the negative feedback inhibition mechanism of serotonergic neurotransmission thereby accelerating and enhancing its antidepressant and anti-anxiety response clinically by bypassing the serotonergic autoreceptors desensitization phenomenon/effects. This effect is achieved as a fast disinhibition process coupled with increased outflow of generated serotonergic neurotransmission action potential from the somatodendritic region toward the terminal presynaptic membrane region which leads to increase serotonin neurotransmitter release (Mcintyre et al., 2017; Gartlehner et al., 2016). While the details of NET-inhibition pharmacodynamics action for SNRIs (that is, noradrenergic neurotransmission enhancing effects) is somehow entirely different from the pharmacoactivity phenomenon that took place in the serotonergic neurotransmission system explained earlier above. This detail explanation on the NET-inhibition mechanism of action for SNRIs will be thoroughly elucidated and unravelled under the selective norepinephrine reuptake inhibitors (NRIs) class of antidepressants described below.

### 4.4 Selective norepinephrine reuptake inhibitors (NRIs)

Reboxetine, viloxazine, teniloxazine (also known as sulfoxazine or sufoxazine), and atomoxetine belong to the selective norepinephrine reuptake inhibitors (NRIs) class of antidepressants. At moderate dose, the NRIs selectively inhibit the norepinephrine reuptake transporters (NET) located at the terminal presynaptic membranes of noradrenergic nerves in the CNS there by leading to a more selective accumulation of norepinephrine within the synaptic clefts. But at high to very high dose, there are postulations that the NRIs may also significantly inhibit dopamine reuptake via NET (as the bupropion-like actions progressively kick-in), especially in areas of the brain such as the prefrontal cortex (neocortex) that are significantly lacking dopamine reuptake transporters (DAT) (Mcintyre et al., 2017; Gartlehner et al., 2016). The pharmacological activity that occurs in the noradrenergic neurotransmission system (mainly at the Locus coeruleus) following chronic administration of NET inhibitors (NRIs or NDRI or SNRIs or TCAs) is somewhat more complex and differs from the pharmacoactivity that happens in the serotonergic neurotransmission systems (mainly at the Raphe nucleus) following chronic administration of SERT inhibitors (SSRIs, SNRIs or TCAs). Acute NET inhibition results in a rapid decline in norepinephrine turnover, as reflected by a fall in concentration of 3-methoxy-4-hydroxyphenylglycol (MHPG), a metabolite of norepinephrine, and a subsequent reduction in the firing rate of the noradrenergic neurons. This effect appears to be mediated by the presynaptic somatodendritic α_2_-adrenergic autoreceptors, which provides inhibitory (negative) feedback mechanism to the presynaptic neuron. In contrast to the serotonergic system, the firing rate of noradrenergic neurons remains inhibited with chronic antidepressant treatment with NRIs or NDRI or SNRIs or TCAs, suggesting that presynaptic somatodendritic α_2_-autoreceptors do not desensitize (i.e. resistant to desensitization phenomenon). However, NET inhibitors (NRIs or NDRI or SNRIs or TCAs) do increase norepinephrine concentrations at postsynaptic sites such as the hippocampus and prefrontal cortex. This indicates desensitization of presynaptic terminal α_2_- autoreceptors (Mcintyre et al., 2017; Kudlow et al., 2012; Rush et al., 2011). Furthermore, chronic antidepressant treatment with reboxetine or buproprion or milnacipram or Amitriptylline results in downregulation or decrease density of postsynaptic β_1_-adrenergic receptors. Research evidence suggests that postsynaptic β_1_-adrenergic receptor downregulation is more likely a key compensatory change. Overall, chronic administration of a NET inhibitor appears to override the downregulation of the postsynaptic β_1_-receptor, resulting in enhanced noradrenergic neurotransmission activity in the central nervous system (CNS). This effect is manifested at the molecular level as increased and enhanced formation of the second intracellular messenger cyclic adenosine monophosphate (cAMP). The postsynaptic β_1_-receptors are Gs protein coupled receptors that activate adenylyl cyclase with resultant conversion of Adenosine Triphosphate (ATP) to cAMP within the inner cell membrane. Alternatively, some of the actions of NET inhibitors are also mediated by the postsynaptic α_1_-adrenoceptors, which do not appear to be downregulated during chronic treatment with the NET inhibitors. The net effect of NET inhibitors on the noradrenergic neurotransmission during chronic treatment is further complicated by regional differences in the distribution of postsynaptic α_1_- and β_1_-adrenergic receptors within the CNS. Nevertheless, the antidepressant effects of NET inhibitors (NRIs, NDRI, SNRIs and TCAs) are being mediated by norepinephrine because inhibition of catecholamine synthesis with α-methyl-p-tyrosine (AMPT) results in the relapse of depression symptoms. They are use for the treatment of major depression, although they have also been used off-label for panic disorder, attention deficit hyperactivity disorder (ADHD), bulimia nervosa, narcolepsy, and treating therapy-resistant paediatric nocturnal enuresis. They are approved for use in many countries worldwide including the United Kingdom, but have not been approved for depression treatment in the United States. Although their effectiveness as an antidepressant has been challenged in multiple published reports, still their popularity has continued to increase (Mcintyre et al., 2017; Gartlehner et al., 2016).

### 4.5 New serotonin receptors antagonist with serotonin reuptake inhibition (SARI)

The SARI class of antidepressant agents include nefazodone, trazodone, and vortioxetine. They exhibit the pharmacological property of a moderate to strong serotonin receptor(s) antagonism with a weak serotonin reuptake transporter (SERT) inhibition, so their primary pharmacodynamics effects and mechanisms of action are not due to SERT inhibition. Nefazodone and trazodone are the pioneer members of this class with potently strong 5-HT_2_ receptor antagonism/blockade (Szegedi et al., 2009; Reynolds et al., 2011).

Vortioxetine is a newer member agent of the SARI class. Some reference literatures refer to vortioxetine as a "serotonin modulator and stimulator" because of its various and diverse pharmacodynamics actions at different serotonergic receptors. It has been shown to possess the following pharmacological activities: namely an antagonist of the 5-HT_3_, 5-HT_7_, and 5-HT_1D_ receptors, a partial agonist of the 5-HT_1B_ receptor, an agonist of the 5HT_1A_ receptor, and a weak inhibitor of the serotonin reuptake transporter (SERT) but its actions are not primarily due to the weak SERT inhibition and it is therefore not classified as an SSRI (Mcintyre et al., 2017; Gartlehner et al., 2016). It has no active metabolites (i.e., it is not a prodrug) and has demonstrated efficacy on major depression in a number of controlled clinical studies. In addition, there are some clinically significant evidences that the drug also improves some aspects of cognition in depressed patients possibly due to its somatodendritic 5-HT_7_ autoreceptors blockade pharmacoactivity. Unfortunately, vortioxetine has no pharmacoactivity at the serotonergic 5-HT_2_ receptor which makes it unique from nefazodone and trazodone with respect to its mechanisms of action (Mcintyre et al., 2017; Gartlehner et al., 2016).

### 4.6 Serotonin 5-HT_1A_ autoreceptor partial agonist with serotonin reuptake inhibition (SPARI)

Vilazodone is the only clinically available member agent of the SPARI class. It was approved in 2011 by the FDA for use in the United States to treat major depressive disorder. It has a multi-ring structure that allows it to exhibit its pharmacological activities. In some ways, its activity can be conceptualized as a combination of an SSRI and buspirone, i.e vilazodone acts as a serotonin reuptake inhibitor with partial agonist activity at the somatodendritic serotonergic 5-HT_1A_ autoreceptors. According to two eight-week, randomized, double-blind, placebo-controlled trials in adults, vilazodone elicits an antidepressant response after one week of treatment. After eight weeks, subjects assigned to vilazodone 40 mg daily dose (titrated over two weeks) experienced a higher response rate than the group given placebo (44% vs 30%, P = 0.002) but the remission rates for vilazodone were not significantly different compared to placebo (Mcintyre et al., 2017; Gartlehner et al., 2016). The partial agonism of somatodendritic serotonergic 5-HT_1A_ autoreceptor by vilazodone in the presence of its SSRI-like activity will enhance and produce fast disinhibition of the serotonergic neurotransmission signals from the midbrain raphe toward the prefrontal cortex, hippocampus and mesolimbic cortex, basal ganglia, and hypothalamus to mediate its respective therapeutic actions in depressive disorders, panic disorder, obsessive-compulsive disorder, and binge-eating disorder (bulimia nervosa); as the somatodendritic serotonergic 5-HT_1A_ autoreceptor desensitization phenomenon has been bypassed by the partial agonistic (weak mixed agonistic-antagonistic) effect in the presence of its SSRI-like activity.

### 4.7 Atypical antipsychotics

The atypical antipsychotics exhibit weak D_2_ receptor antagonism with potently strong 5-HT_2A/2C_ receptor blockade (or inverse agonism). In most cases, they also act as partial agonists at the 5-HT_1A_ autoreceptor, which produces synergistic effects with the 5-HT_2A/2C_ receptor antagonism. Most atypical antipsychotics are either 5-HT_6_ or 5-HT_7_ receptor antagonists. Atypical antipsychotics such as olanzapine, quetiapine, clozapine, risperidone, lurasidone, aripiprazole and brexpiprazole are now being used by clinical psychiatrists as a sole or adjunct-augmenting pharmacotherapeutic agent in the management of major depressive disorder (MDD) that has been unresponsive or showed inadequate remission after 4-8 weeks of active treatment with other classes of antidepressants such as the SSRIs, SNRIs, or TCAs (Mcintyre et al., 2017; Gartlehner et al., 2016).

Aripiprazole and its new structural congener brexpiprazole exhibit partial agonist activity (that is, weak mixed agonist-antagonist action) at both the dopaminergic D_2_ and serotonergic 5-HT_1A_ receptors but still maintain a potently strong 5-HT_2A/2C_ receptor blockade (or inverse agonism). An atypical antipsychotic agent will potently block or antagonize the postsynaptic serotonergic 5-HT_2A_ and 5-HT_2C_ receptors in the prefrontal cortex to mediate its antidepressant effect clinically. While the synergistic influence/combination of the full antagonism of the somatodendritic serotonergic 5-HT_7_ autoreceptor by an atypical antipsychotic and the partial agonism of the somatodendritic serotonergic 5-HT_1A_ autoreceptor by an atypical antipsychotic in the presence of an SSRI or SNRI or NASSA or TCA will enhance and produce fast disinhibition of the serotonergic neurotransmission signals from the midbrain raphe nucleus toward the prefrontal cortex to mediate moderately quick-onset antidepressant action within 2-4 weeks of administration as the somatodendritic serotonergic 5-HT_7_ and 5-HT_1A_ autoreceptors desensitization phenomenon has been bypassed by the full antagonistic and the partial agonistic (weak mixed agonistic-antagonistic) effects of these two autoreceptors, respectively. Furthermore, by antagonizing the neocortical postsynaptic serotonergic 5-HT_2C_ receptors on the noradrenergic and dopaminergic neurotransmission pathways in the prefrontal cortex, an atypical antipsychotics disinhibits/increases norepinephrine and dopamine release specifically in the neocortical areas such as the prefrontal cortex but neither in the subcortical areas such as the basal ganglia, hippocampus nor mesolimbic cortex. Therefore, an atypical antipsychotics is a norepinephrine–dopamine disinhibitor (NDD). The mesolimbic cortex comprises of dopaminergic neuronal projections from the ventral tegmental area toward the nucleus accumbens shell. It also worth mentioning here that dopaminergic and noradrenergic neurotransmission pathways in neocortical areas such as the prefrontal cortex, entorrhinal cortex, cingulate cortex, superior temporal cortex and orbital cortex are hypofuctionally impaired in depressive disorders. Infact, it has been demonstrated that genetically modified knock-out experimental model mice lacking 5-HT_2A_ and/or 5-HT_2C_ receptors significantly exhibits/manifests reduced and limited anxiety symptoms. Hence, by antagonizing the postsynaptic serotonergic 5-HT_2A_ and 5-HT_2C_ receptors in the subcortical areas such as basal ganglia, mesolimbic cortex and hippocampus; an atypical antipsychotic will produce anxiolytic effect clinically. The synergistic influence/combination of the full antagonism of the somatodendritic serotonergic 5-HT_7_ autoreceptor by an atypical antipsychotic and the partial agonism of the somatodendritic serotonergic 5-HT_1A_ autoreceptor by an atypical antipsychotic in the presence of an SSRI or SNRI or NASSA or TCA will also enhance and produce fast disinhibition of the serotonergic neurotransmission signals from the midbrain raphe nucleus toward the hippocampus and mesolimbic cortex, basal ganglia, and hypothalamus to mediate its respective therapeutic actions in panic disorder, obsessive-compulsive disorder, and binge-eating disorder (bulimia nervosa). As clinical findings and evidences support the interference of an atypical antipsychotic with the different serotonergic neurotransmission pathways mediating and controlling different neuropsychiatric disorders. In each case, an atypical antipsychotic induced disinhibition of serotonergic neurotransmission with delivering of serotonin neurotransmitter where it is needed, hypothetically in different neocortical and subcortical areas for different neuropsychiatric disorders. Clinical observations obviously support the fact that different serotonergic pathways mediate the different therapeutic actions of an atypical antipsychotic, since pharmacological actions on different neocortical and subcortical areas depend on which particular neuropsychiatric disorder is being therapeutically targeted. From the psychopharmacological point of view, an atypical antipsychotic will be efficacious as a sole monotherapy or adjunct-augmenting pharmacotherapeutic agent for the treatment of patients having anxious depression disorders (that is, either major depressive disorder [MDD] or bipolar depression or schizoaffective/psychotic depression with anxiety disorder component). The atypical antipsychotics appear to be more consistently effective in the treatment of bipolar depression and also do not increase the risk of inducing mania or increasing the frequency of bipolar cycling. Infact patients with depression disorders tend to even respond far better and become clinically more stable (undergo remission faster) on an atypical antipsychotic alone as monotherapy compare to the other old conventional antidepressant agents such as TCA, SSRI or SNRI alone (Gerhard et al., 2014; Sheffrin et al., 2009; Thase et al., 2015). This is one of the main reasons behind FDA approval of a fixed dose combination of an SSRI with an atypical antipsychotic such as Fluoxetine and Olanzapine. A fixed dose combination of an SSRI with an atypical antipsychotic such as Fluoxetine and Olanzapine has received FDA approval for the pharmacotherapy of major depressive disorder (MDD), acute bipolar depression, and schizoaffective (psychotic) depression. Also a fixed dose combination of Sertraline and Aripiprazole is currently undergoing clinical trial investigation for the same indications. The 5-HT_1B/1D_ and 5-HT_1A_/5-HT_7_ autoreceptors play important roles in regulating the terminal presynaptic release of serotonin neurotransmitter and the somatodendritic onset depolarizing activity of serotonergic neurons respectively. Increase serotonin neurotransmitter at the somatodendritic region and the terminal presynaptic region, which is as a result of an SSRI or SNRI or NASSA or TCA pharmacodynamics effect activates the combinations of the somatodendritic serotonergic 5-HT_1A_/5-HT_7_ autoreceptors and the terminal presynaptic serotonergic 5-HT_1B/1D_ autoreceptors thereby leading to a decrease in the firing rate at the somatodendritic region and a decrease in serotonin neurotransmitter release from the terminal presynaptic region, respectively, thus reducing the antidepressant and the anxiolytic clinical responses (sensitization or pre-desensitization phenomenon/effects). Furthermore, it also worth mentioning here that the addition of a drug like a selective 5-HT_7_ autoreceptor antagonist with 5-HT_1A_ autoreceptor partial agonism (such as an atypical antipsychotic); or alternatively a selective 5-HT_1A_ autoreceptor partial agonist (such as buspirone or tandospirone); or alternatively a selective 5-HT_1A_ and 5-HT_1B/1D_ autoreceptors antagonist (such as pindolol) to an SSRI or SNRI or NASSA or TCA treatment will decouple the negative feedback inhibition mechanism of serotonergic neurotransmission thereby accelerating and enhancing its antidepressant and anxiolytic response clinically by bypassing the serotonergic autoreceptors desensitization phenomenon/effects. This effect is achieved as a fast disinhibition process coupled with increase outflow of different generated serotonergic neurotransmission action potentials from the different somatodendritic regions at the midbrain raphe nucleus toward the different terminal presynaptic membrane regions located at different cortical- (prefrontal cortex) and subcortical- (hippocampus, mesolimbic cortex, basal ganglia and hypothalamus) areas of the brain to mediate the observed therapeutic effects in different neuropsychiatric disorders due to increase serotonin neurotransmitter release.

The pro-serotonergic neurotransmission enhancing activity of an atypical antipsychotic in the absence or presence of an SSRI or SNRI or NASSA or TCA is completely antagonized and diverted away from the somatodendritic serotonergic 5-HT_1A_ and 5-HT_7_ autoreceptors, and postsynaptic serotonergic 5-HT_1A_ , 5-HT_2A_ , 5-HT_2C_ , 5-HT_6_ and 5-HT_7_ receptors toward the other terminal presynaptic- and postsynaptic-serotonergic subtype receptors in the central nervous system (CNS). Lastly, antidepressant and anxiolytic activities can arise through this novel mechanism of action as in the case of atypical antipsychotics (Mcintyre et al., 2017; Gartlehner et al., 2016; Gerhard et al., 2014; Sheffrin et al., 2009; Thase et al., 2015; Rutherford et al., 2007).

What this review adds to the body of knowledge

• The emerging antidepressants are: selective monoamine oxidase inhibitors (MAOIs) such as bifemelane, pirlindole, toloxatone, selegiline, rasagiline and safinamide; serotonin-norepinephrine reuptake inhibitors (SNRIs) such as ansofaxine, nefopam and levomilnacipran; norepinephrine reuptake inhibitors (NRIs) such as Reboxetine, viloxazine, teniloxazine (also known as sulfoxazine or sufoxazine), and atomoxetine; Vilazodone (SPARI); Vortioxetine (SARI); atypical antipsychotics such as olanzapine, quetiapine, risperidone, lurasidone, aripiprazole and brexpiprazole; N-methyl-d-aspartate (NMDA)-glutamatergic neurotransmission system blockers such as ketamine, CP-101,606 (traxoprodil), GLYX-13 (rapastinel), NRX-1074 (Apimostinel) and Riluzole. While Agomelatine (MASSA) remains a paradoxical agent that doesn't fit into any of the currently available classes of antidepressant agents and its pharmacological properties also deem it unfit and inappropriate to be classified into another separate novel class of antidepressants contrary to the reports published in previous reference literatures.
• More proactive research should be done to synthesize rapid-onset novel antidepressant agents that will act selectively on the N-methyl-d-aspartate (NMDA)-glutamatergic ionoceptor as an antagonist or inverse agonist or partial agonist without producing the neurocognitive dysfunction, dissociative, and psychotomimetic (hallucinogenic) effect associated with the blockade of this receptor.
• From the psychopharmacological point of view, agomelatine will be efficacious as an adjunct-augmenting pharmacotherapeutic agent for the treatment of patients having anxious depression disorders (that is, either major depression disorder [MDD] or bipolar depression or schizoaffective depression with anxiety disorder component). It will also be efficacious as a sole or combine pharmacotherapeutic agent for the treatment of patients having delayed sleep phase syndrome due to circadian rhythm desynchronisation disorder type 1 (CRDD-1) or Jetlag dysrhythmia, insomnia, anxiety disorders, selective serotonin reuptake inhibitor (SSRI)-induced sexual dysfunction and/or SSRI-induced nocturnal myclonus/akathisia.
• Agomelatine alone may not be effective as a monotherapy for the treatment of unipolar depression or bipolar depression or schizoaffective depression because of its unique mechanism of action as a melatonergic MT**_1_** and MT**_2_** receptors agonist and a selective serotonergic 5-HT**_2C_** receptor antagonist (MASSA).
• Because Agomelatine lacks inhibitory pharmacoactivity at the monoaminergic reuptake transporter pumps (SERT, NET and DAT), does not inhibit the enzyme monoamine oxidase, has neither weak antagonist nor partial agonist activity at the dopaminergic D**_2_** receptor, and also lacks antagonistic activity at both the noradrenergic α_2_ -receptor and N-methyl-d-aspartate (NMDA)-glutamatergic ionoceptor; it should not be regarded and accepted as an antidepressant but rather it should be classified as an anxiolytic-sedative agent on account of its melatonergic MT**_1_** and MT**_2_** receptors agonist and selective serotonergic 5-HT**_2C_** receptor antagonistic (MASSA) properties.
• This review remarkably advocates for the incorporation of the atypical antipsychotics and N-methyl-d-aspartate (NMDA)-glutamatergic ionoceptor blockers as new member classes of the antidepressant agents because of their clinically significant roles in the management of depression disorders.
• Vilazodone is currently the only clinically available member agent that belongs to the pharmacologically distinct and unique class SPARI.
• Currently, ketamine is a better inexpensive, less strenuous and more effective substitute for Electroconvulsive therapy (ECT) in the management of treatment-resistant MDD or bipolar depression or schizoaffective depression. Both sub-anaesthetic low dose of ketamine and ECT produced antidepressant effects; however, ketamine produced superior antidepressant effects in terms of fast response onset. For example, ketamine produced rapid antidepressant effects starting at 24 h; whereas, the antidepressant effects of ECT were not expressed until after 48 h. The antidepressant effects of both ketamine and ECT lasted for at least seven days. This shows that ketamine is more efficacious than ECT for treating MDD or bipolar depression or schizoaffective depression.
• The new evolving potential drug targets for depression treatment are the NMDA-glutamatergic receptor as antagonist or inverse agonist or partial agonist; metabotropic glutamatergic receptors as positive or negative modulator; excitatory amino acid transporter-2 (EAAT-2) as a reuptake enhancer; and as a terminal presynaptic glutamate release inhibitor.

## 5 Conclusion

The emerging antidepressants are: selective monoamine oxidase inhibitors (MAOIs) such as bifemelane, pirlindole, toloxatone, selegiline, rasagiline and safinamide; serotonin-norepinephrine reuptake inhibitors (SNRIs) such as ansofaxine, nefopam and levomilnacipran; norepinephrine reuptake inhibitors (NRIs) such as Reboxetine, viloxazine, teniloxazine (also known as sulfoxazine or sufoxazine), and atomoxetine; Vilazodone (SPARI); Vortioxetine (SARI); atypical antipsychotics such as olanzapine, quetiapine, risperidone, lurasidone, aripiprazole and brexpiprazole; N-methyl-d-aspartate (NMDA)-glutamatergic neurotransmission system blockers such as ketamine, CP-101,606 (traxoprodil), GLYX-13 (rapastinel), NRX-1074 (Apimostinel) and Riluzole. While Agomelatine (MASSA) remains a paradoxical agent that doesn't fit into any of the currently available classes of antidepressant agents and its pharmacological properties also deemed it unfit and inappropriate to be classified into another separate novel class of antidepressants contrary to the reports published in previous reference literatures. More proactive research should be done to synthesize rapid-onset novel antidepressant agents that will act selectively on the N-methyl-d-aspartate (NMDA)-glutamatergic ionoceptor as an antagonist or inverse agonist or partial agonist without producing the neurocognitive dysfunction, dissociative, and psychotomimetic (hallucinogenic) effect associated with the blockade of this receptor. Lastly, this review remarkably advocates for the incorporation of the atypical antipsychotics and N-methyl-d-aspartate (NMDA)-glutamatergic ionoceptor blockers as new member classes of the antidepressant agents because of their clinically significant roles in the management of depression disorders.

## Grants and financial support

Nil.

## Conflicts of interest

The author of this review declares that there is no conflict of interest.

## References

[bib0005] Auer D.P., Pütz B., Kraft E., Lipinski B., Schill J., Holsboer F. (2000). Reduced glutamate in the anterior cingulate cortex in depression: an in vivo proton magnetic resonance spectroscopy study. Biol. Psychiatry.

[bib0010] Autry A.E., Adachi M., Nosyreva E., Na E.S., Los M.F., Peng-fei C. (2011). Monteggia LM. NMDA receptor blockade at rest triggers rapid behavioural antidepressant responses. Nature.

[bib0015] Azbill R.D., Mu X., Springer J.E. (2000). Riluzole increases high-affinity glutamate uptake in rat spinal cord synaptosomes. Brain Res..

[bib0020] Beneyto M., Meador-Woodruff J.H. (2008). Lamina-specific abnormalities of NMDA receptor-associated postsynaptic protein transcripts in the prefrontal cortex in schizophrenia and bipolar disorder. Neuropsychopharmacology.

[bib0025] Beneyto M., Kristiansen L.V., Oni-Orisan A., McCullumsmith R.E., Meador-Woodruff J.H. (2007). Abnormal glutamate receptor expression in the medial temporal lobe in schizophrenia and mood disorders. Neuropsychopharmacology.

[bib0030] Berman R.M., Cappiello A., Anand A., Oren D.A., Heninger G.R., Charney D.S., Krystal J.H. (2000). Antidepressant effects of ketamine in depressed patients. Biol. Psychiatry.

[bib0035] Berman R.M., Sanacora G., Anand A., Roach L.M., Fasula M.K., Finkelstein C.O. (2002). Charney DS. Monoamine depletion in unmedicated depressed subjects. Biol. Psychiatry.

[bib0040] Cipriani A., Furukawa T.A., Salanti G., Chaimani A., Atkinson L.Z., Ogawa Y. (2018). Comparative efficacy and acceptability of 21 antidepressant drugs for the acute treatment of adults with major depressive disorder: a systematic review and network meta-analysis. Lancet.

[bib0045] Dale Elena, Bang-Andersen Benny, Sánchez Connie (2015). Emerging mechanisms and treatments for depression beyond SSRIs and SNRIs. Biochem. Pharmacol..

[bib0050] Fasipe O.J., Osho P.O., Osho E.S., Akhideno P.E., Ibiyemi-Fasipe O.B. (2018). Agomelatine repositioning as a novel anxiolytic-sedative agent for the treatment of circadian rhythm desynchronisation disorder type 1, insomnia and anxiety disorders in clinical practice. The Society Francophone of Chronobiology (SFC) Book of Abstracts and Conference Proceedings, 2018;76. Research Presentation at the 46th Conference of the Society Francophone of Chronobiology (SFC), Which Took Place at the Hassan II Institute of Agronomy and Veterinary Medicine.

[bib0055] Gartlehner G., Gaynes B.N., Amick H.R. (2016). Comparative benefits and harms of antidepressant, psychological, complementary, and exercise treatments for major depression: an evidence report for a clinical practice guideline from the American College of Physicians. Ann. Intern. Med..

[bib0060] Gelenberg A.J., Freeman M.P., Markowitz J.C. (2010). Practice Guideline for the Treatment of Patients With Major Depressive Disorder.

[bib0065] Gerhard T., Akincigil A., Correll C.U., Foglio N.J., Crystal S., Olfson M. (2014). National trends in second-generation antipsychotic augmentation for nonpsychotic depression. J. Clin. Psychiatry.

[bib0070] Ghasemi M., Kazemi M.H., Yoosefi A., Ghasemi A., Paragomi P., Amini H., Afzali M.H. (2014). Rapid antidepressant effects of repeated doses of ketamine compared with electroconvulsive therapy in hospitalized patients with major depressive disorder. Psychiatry Res..

[bib0075] Heun R., Coral R.M., Ahokas A., Nicolini H., Teixeira J.M., Dehelean P. (2013). 1643 – Efficacy of agomelatine in more anxious elderly depressed patients. A randomized, double-blind study vs placebo. Eur. Psychiatry.

[bib0080] Kasper S., Hajak G., Wulff K., Hoogendijk W.J., Montejo A.L., Smeraldi E., Rybakowski J.K., Quera-Salva M.A., Wirz-Justice A.M., Picarel-Blanchot F., Baylé F.J. (2010). Efficacy of the novel antidepressant agomelatine on the circadian rest-activity cycle and depressive and anxiety symptoms in patients with major depressive disorder: a randomized, double-blind comparison with sertraline. J. Clin. Psychiatry.

[bib0085] Koesters M., Guaiana G., Cipriani A., Becker T., Barbui C. (2013). Agomelatine efficacy and acceptability revisited: systematic review and meta-analysis of published and unpublished randomised trials. Br. J. Psychiatry.

[bib0090] Kudlow P.A., Cha D.S., McIntyre R.S. (2012). Predicting treatment response in major depressive disorder: the impact of early symptomatic improvement. Can. J. Psychiatry.

[bib0095] Lally N., Nugent A.C., Luckenbaugh D.A., Ameli R., Roiser J.P., Zarate C.A. (2014). Anti-anhedonic effect of ketamine and its neural correlates in treatment-resistant bipolar depression. Transl. Psychiatry.

[bib0100] Lapidus K.A.B., Levitch C.F., Perez A.M., Brallier J.W., Parides M.K., Soleimani L., Murrough J.W. (2014). A randomized controlled trial of intranasal ketamine in major depressive disorder. Biol. Psychiatry.

[bib0105] Lara D.R., Bisol L.W., Munari L.R. (2013). Antidepressant, mood stabilizing and procognitive effects of very low dose sublingual ketamine in refractory unipolar and bipolar depression. Int. J. Neuropsychopharmacol..

[bib0110] Mcintyre R.S., Suppes T., Tandon R., Ostacher M.J. (2017). Florida Best Practice Psychotherapeutic Medication Guidelines for adults with major depressive disorder. J. Clin. Psychiatry.

[bib0115] Panos Z., Moaddel R., Morris P.J., Georgiou P., Fischell J., Elmer G.I. (2016). NMDAR inhibition-independent antidepressant actions of ketamine metabolites. Nature.

[bib0120] Reynolds C.F.I.I.I., Butters M.A., Lopez O. (2011). Maintenance treatment of depression in old age: a randomized, double blind, placebo-controlled evaluation of the efficacy and safety of donepezil combined with antidepressant pharmacotherapy. Arch. Gen. Psychiatry.

[bib0125] Rush A.J., Trivedi M.H., Stewart J.W. (2011). Combining medications to enhance depression outcomes (CO-MED): acute and long-term outcomes of a single-blind randomized study. Am. J. Psychiatry.

[bib0130] Sanacora G., Kendell S.F., Levin Y., Simen A.A., Fenton L.R., Coric V., Krystal J.H. (2007). Preliminary evidence of riluzole efficacy in antidepressant-treated patients with residual depressive symptoms. Biol. Psychiatry.

[bib0135] Sheffrin M., Driscoll H.C., Lenze E.J. (2009). Pilot study of augmentation with aripiprazole for incomplete response in latelife depression: getting to remission. J. Clin. Psychiatry.

[bib0140] Stein D.J., Picarel-Blanchot F., Kennedy S.H. (2013). Efficacy of the novel antidepressant agomelatine for anxiety symptoms in major depression. Hum. Psychopharmacol. Clin. Exp..

[bib0145] Szegedi A., Jansen W.T., van Willigenburg A.P., van der Meulen E., Stassen H.H., Thase M.E. (2009). Early improvement in the first 2 weeks as a predictor of treatment outcome in patients with major depressive disorder: a meta-analysis including 6562 patients. J. Clin. Psychiatry.

[bib0150] Thase M.E., Youakim J.M., Skuban A. (2015). Efficacy and safety of adjunctive brexpiprazole 2 mg in major depressive disorder: a phase 3, randomized, placebo-controlled study in patients with inadequate response to antidepressants. J. Clin. Psychiatry.

[bib0155] Wray N.H., Schappi J.M., Singh H., Senese N.B., Rasenick M.M. (2018). NMDAR-independent, cAMP-dependent antidepressant actions of ketamine. Springer Nat. J. Mol. Psychiatry.

[bib0160] Yamakura T., Shimoji K. (1999). Subunit- and site-specific pharmacology of the NMDA receptor channel. Prog. Neurobiol..

[bib0165] Yamakura T., Mori H., Masaki H., Shimoji K., Mishina M. (1993). Different sensitivities of NMDA receptor channel subtypes to non-competitive antagonists. NeuroReport.

[bib0170] Zarate C.A., Payne J.L., Quiroz J., Sporn J., Denicoff K.K., Luckenbaugh D., Manji H.K. (2004). An openlabel trial of riluzole in patients with treatment-resistant major depression. Am. J. Psychiatry.

[bib0175] Zarate C.A., Mathews D., Ibrahim L., Chaves J.F., Marquardt C., Ukoh I., Luckenbaugh D.A. (2013). A randomized trial of a low-trapping nonselective N- Methyl-D-Aspartate channel blocker in major depression. Biol. Psychiatry.

[bib0180] Zigman D., Blier P. (2013). Urgent ketamine infusion rapidly eliminated suicidal ideation for a patient with major depressive disorder: a case report. J. Clin. Psychopharmacol..

